# Micellization of Organic Conjugated Polymers toward Functional Nanomaterials for Photocatalytic Applications

**DOI:** 10.1021/cbe.5c00079

**Published:** 2026-01-14

**Authors:** Feng Qiu, Ke Huang, Peng Tao, Sheng Han, Wai-Yeung Wong

**Affiliations:** † School of Chemical and Environmental Engineering, 92263 Shanghai Institute of Technology, 100 Haiquan Road, Shanghai 201418, P. R. China; ‡ Department of Applied Biology and Chemical Technology and Research Institute for Smart Energy, 26680 The Hong Kong Polytechnic University, Hung Hom, Hong Kong 999077, P. R. China

**Keywords:** conjugated polymer, micellization, photosensitizer, electron/energy transfer, photocatalyst

## Abstract

Organic conjugated polymers (CPs) with an extended π-conjugated backbone exhibiting unique photophysical and chemical properties (e.g., good chemical stability, tunable light absorption, superior charge mobility, etc.) have been developed as promising photosensitizers for wide photocatalytic applications. Most of the reviews concerning CPs for photocatalytic applications focus on the structural design of CPs to achieve unique photophysical properties including broad absorption spectrum, high molar absorption coefficient, and low photobleaching. Construction of CPs into porous topological structures provides rich active sites. Moreover, the crystallinity of CPs facilitates photoinduced exciton transport across the polymeric backbone. However, the inherent hydrophobic character of these CPs exhibits low solubility in aqueous conditions, resulting in the existence of photoinduced exciton recombination, which would limit their photocatalytic applications. Micellization of CPs with hydrophilic functional groups via covalent/noncovalent interactions to construct the conjugated polymeric nanomaterials (CPNs) with improved surface wettability is an efficient approach to the construction of high-performance photocatalysts, resulting in their high dispersion stability in an aqueous solution and enhanced electron transfer from the photocatalyst to the reactant by decreasing their interfacial free energy. In this perspective, we provide a critical overview of the recent progress on the design and synthesis of the CPNs, focusing particularly on the relationship between the morphology and size of CPNs and their optoelectronic properties. Moreover, their photocatalytic applications including organic pollutant degradation, photoinduced chemical reactions, hydrogen evolution, CO_2_ fixation, and medical therapy are discussed systematically. The current challenges and perspectives of CPNs for photocatalytic applications are also highlighted.

## Graphical abstract

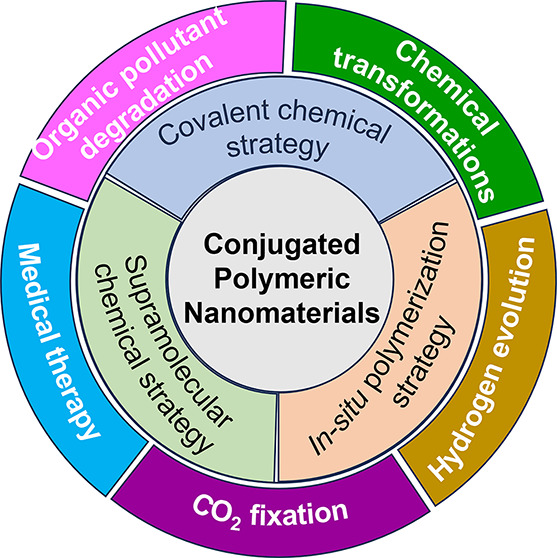

## 1 Introdution

The success of chemical reactions with the generation of products often requires overcoming the energy barrier in reaction systems by increasing reaction temperature. To minimize this energy barrier, various chemical catalysts have been well developed. However, the energy consumption from natural resources cannot be avoided.^1,2^ With the emerging and threatening of energy shortages and air pollution, the demand for green chemical reaction under a mild reaction condition has received great attention in both academic and industrial communities.^3^ To date, mild reaction techniques such as bioenzymatic, electrocatalytic, and photocatalytic reactions have been exploited for wide applications in chemical, materials, and biological science.^4–6^ Among these technologies, the photocatalytic reaction mimicking the natural synthesis is a mature strategy for chemical generation with many advantages, including the direct usage of solar energy, mild reaction condition with low temperature and normal pressure, less side reaction, high stability, and broad applications.^7,8^ With the advancements in nanotechnology, catalytic reactive materials have been extensively developed, making photocatalytic applications increasingly promising in the fields of materials chemistry, new energy conversion, and biomedicine, while contributing to the reduction of traditional energy consumption.^9,10^ Thus, the design and construction of photocatalysts with high sensitivity and excellent specificity to afford promising photochemical behavior remains a great challenge.

In the last two decades, inorganic materials usually show the best photocatalytic properties for energy transfer and conversion and have been well investigated because of their good semiconductivity, biocompatibility, and low cost.^11^ For example, titanium dioxide (TiO_2_) has been reported as the most efficient oxidative photocatalyst for the microbiocidal disinfection effects in 1985.^12^ Then, TiO_2_ was also found to show high photoactivity for organic pollutant degradation, hydrogen (H_2_) generation, carbon dioxide (CO_2_) reduction, nitrogen (N_2_) fixation, and so on.^13^ With the increasing demand of photocatalytic applications, many other transition-metal oxides/sulfides/carbides (MoS_2_, ZnO, and BiOX (X = Cl, Br, and I), etc.) have been developed as the photocatalysts with unique performances.^14–16^ Moreover, various strategies (e.g., heteroatom-doping approach, defect engineering approach, and formation of the heterostructure system with controlled core–shell or hollow nanostructures) have been developed to improve their charge generation and separation.^17,18^ Unfortunately, these inorganic materials have their inherent drawbacks including low chemical stability (especially in acid environment), low specific surface area, strong tendency to aggregation, large bandgap energy, and narrow light absorption, severely restricting their practical applications in large-scale production.^19^ In comparison with inorganic materials, organic conjugated materials consisting of multitudinous polycyclic aromatic hydrocarbons (PAHs) are typically extended π-conjugated systems, which endow them with unique photophysical and chemical properties, such as lightweight, good chemical stability, simple preparation process, superior charge mobility, broad absorption region, etc.^20,21^ Furthermore, the incorporation of heteroatoms (e.g., B, N, and S) into the backbone of organic semiconductive materials could efficiently tune their optoelectronic properties, such as molecular energy levels, charge generation, and separation.^22,23^ Therefore, the development of organic conjugated materials as promising photocatalysts has received considerable attention from scientists from a wide range of fields.

As the staring organic photocatalyst, the graphitic carbon nitride (g-C_3_N_4_) possesses a light absorption edge at around 450 nm with a bandgap of ca. 2.7 eV, while the nitrogen-based groups exhibit the efficient catalytic activation sites, enabling it to be the metal-free photocatalyst under simulated sunlight.^24^ Unfortunately, practical applications of bare g-C_3_N_4_ are hindered by its high hole–electron recombination rate, low electrical conductivity, and lack of absorption above 460 nm. It is well-known that the visible light accounts for ∼50% of the energy of solar light, which is much higher than that of the ultraviolet light (7%). To date, scientists have made numerous efforts to develop π-conjugated polymers (CPs) with outstanding visible-light absorption for organic photovoltaic cells, organic light-emitting diodes, and organic field effect transistors.^19,21,25^ The backbone of CPs with a donor–acceptor (D–A) structure can be facilely controlled by changing the type of building blocks, resulting in the tunable absorption spectrum covering from the blue to red light region.^26^ Therefore, these CPs are also demonstrated to be good visible-light active photocatalysts for the wide applications from the environment, chemistry, biology, to energy field. Due to their rigid and hydrophobic architectures, however, CPs still have their weaknesses for photocatalytic applications in the aqueous condition, such as ease of exciton recombination, limited light absorption, and hidden active sites (Scheme 1a).^27^ Construction of covalent organic frameworks (COFs) or metal–organic frameworks (MOFs) can relieve some restrictions of CPs, but harsh reaction conditions and limited choice of monomers are still apparent.

**Scheme 1:**
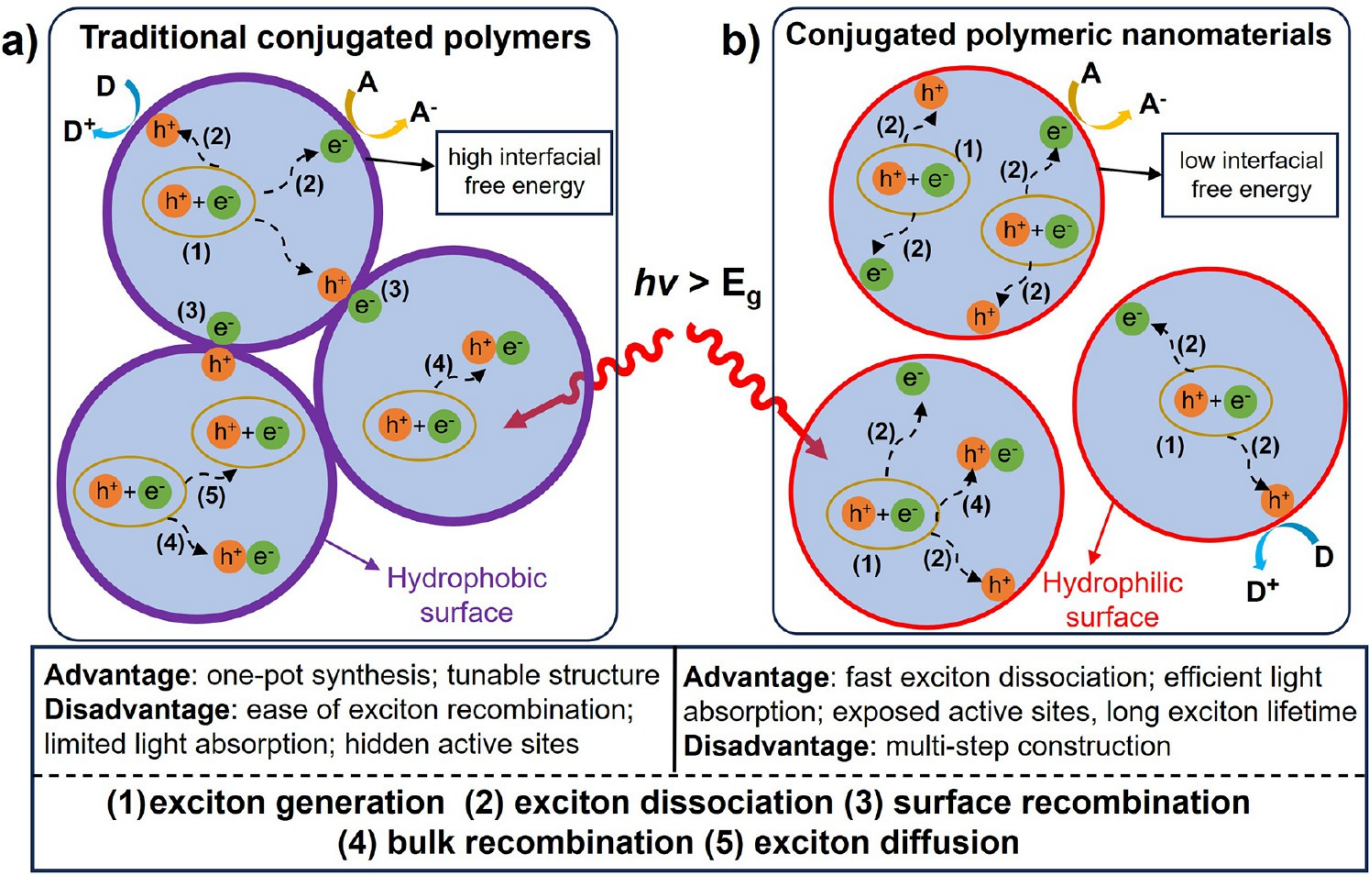
Comparison of the Advantages and Disadvantages of (a) Traditional CPs and (b) Conjugated Polymeric Nanomaterials (CPNs) in Photocatalytic Performance^21^

Micellization is a spontaneous gateway to prepare the nanomaterials in the aqueous condition with controlled morphology and ordered structural arrangement.^28^ Since the 1980s, macromolecular self-assembly of block copolymers into micelles has been well developed and is being extensively explored in the fields of electronic devices, medicine delivery, sensors, microreactors, etc.^29^ The main driving force of the macromolecular self-assembly is the repulsion between unlike blocks and the cohesive interaction between the like blocks to form the phase separation in solution or bulk via one but not limited to one noncovalent interactions like the hydrophobic/hydrophilic effect, host-guest interaction, π–π interactions, dipole–dipole interaction, hydrogen bonding, and so on.^30^ π-Conjugated polymers are a kind of ideal polymeric blocks for macromolecular self-assembly through hydrophobic/hydrophilic and π–π interaction due to their hydrophobic and rigid structure. So far, the development of the self-assembled π-conjugated polymer has provided an effective approach to achieve a variety of functional organic nanomaterials.^31,32^ Owing to the strong π–π interaction in these rigid PAHs, the morphologies with one-dimension or two-dimension of CPNs can be controlled.^33^ The charge transfer and separation in these nanomaterials could also be improved efficiently through the electron hopping between the intermolecular polymeric backbone.^34,35^ In this regard, several key features of the CPNs are received, including fast exciton dissociation, efficient light absorption, exposure to more active sites, and long exciton lifetimes (Scheme 1b).^21,33^ Such outstanding physical and optoelectronic characteristics of CPNs have promoted the remarkable achievements in a broad range of photocatalytic applications.^25,36,37^ In the past years, a tremendous number of polymeric materials have been witnessed for photocatalytic reactions. Even previous reviews are concerned in some branches of CPs for photocatalytic applications, like conjugated porous polymers,^38^ COFs,^39^ and MOFs.^40^ However, a systematic review on the functionalized CPNs for photocatalytic applications in the environment, biology, and chemical conversion has not yet been well documented. In this Review, depending on the various chemical synthetic approaches in these CPNs, we particularly emphasize the design methodologies of CPNs, including a covalent chemical strategy, supramolecular chemical strategy, and in situ polymerization strategy. Besides, the difficulty, micellar size, stability, and scalability in the preparation method of CPNs for photochemical applications are summarized in Table 1. Their photochemical applications for these CPNs in organic pollutant degradation, chemical transformations, hydrogen evolution, CO_2_ fixation, and medical therapy are presented. Finally, the summaries and the future challenges for CPNs in the field of photocatalytic application are also discussed.

**Table 1:** Summary of Difficulty, Micellar Size, Stability, Morphology, and Scalability for the Preparation Methods of CPNs for Photochemical Applications

preparation method	difficulty	size	morphology	stability	scalability
covalent chemical strategy	medium	10 nm to 1 μm	fibers, spheres, 2D layers	medium	medium, hundred milligrams to 1 g
supramolecular chemical strategy	simple	20 nm to 100 nm	spheres, hollows	low	large, higher than 1 g
in situ polymerization strategy	complicated	100 nm to several micrometers	spheres, fibers, bowls	high	low, down to hundred milligrams

## 2 Synthesis of Conjugated Polymeric Nanomaterials

### 2.1 Chemical Strategy by Covalent Functionalization

#### 2.1.1 Side-Chain Covalent Functionalization

With the rapid development of π-conjugated polymers in optoelectronic devices, the preparation of these polymers with the adjusted backbone has been well established via different cross-coupling polymerization, Knoevenagel polycondensation, or oxidative polymerization of aromatic monomers.^41^ Furthermore, the side-chain engineering approach has been verified as the efficient modification method, which not only would improve the solubility and processability of CPs but also could tune the aggregate behavior of CPs and their optical and electronic properties.^42^ Many researchers have introduced the different ionic groups on the side chain of CPs, named conjugated polyelectrolytes (CPEs), which exhibit good performance in photovoltaic cell, organic light emitting diodes, sensors, etc.^43,44^

In 2017, Wang and co-workers prepared sodium carboxylate-modified polythiophene (PT), showing the nanosized aggregates with a hydrodynamic diameter of ∼220 nm in aqueous solution (Figure 1a,b).^45^ Owing to the negative surface potentials of this PT, it could also form the photocatalyst complex with the electron mediator of methyl viologen (MV^2+^) via electrostatic interactions (Figure 1c,d). In addition, this complex can also be bound to self-assembled Aβ_16–22_, resulting in the enhancement of photoinduced electrons transfer from PT to MV^2+^.^46^ The quaternary ammonium ion is another kind of water-soluble group found in a wide range of commercial surfactants. Fluorene bearing tunable side chains is a classical electron-donating conjugated building block for the preparation of CPEs. Thus, Huang and co-workers prepared D–A-type CPEs by copolymerization of quaternary ammonium salt-grafted fluorine with different electron-withdrawing monomers (e.g., 1,4-dicyanobenzene, benzothiadiazole (BT), and pyrrolopyrroledione) (Figure 1e).^47^ These polyelectrolytes could be highly soluble in water/alcohol-like polar solvents. Moreover, their UV–vis absorption spectra and energy levels were adjusted efficiently by the electron-withdrawing capability of acceptor moieties (Figure 1f,g). Similar fluorine-based polymeric materials have also been reported by Liu’s and Lai’s groups, respectively.^48–50^ Furthermore, the group of quaternary ammonium salt grafted on the side chain of CP could be replaced by pyridinium salt (Figure 1h).^51^ The pyridinium moieties can not only provide good water/alcohol solubility but also generate the radical of DPTFBr with the reducing agent of triethanolamine (TEOA) under an illumination (Figure 1i), which was confirmed by the electron spin resonance (ESR) spectra. This radical structure could be recovered by ambient O_2_ (Figure 1j).

**Figure 1:**
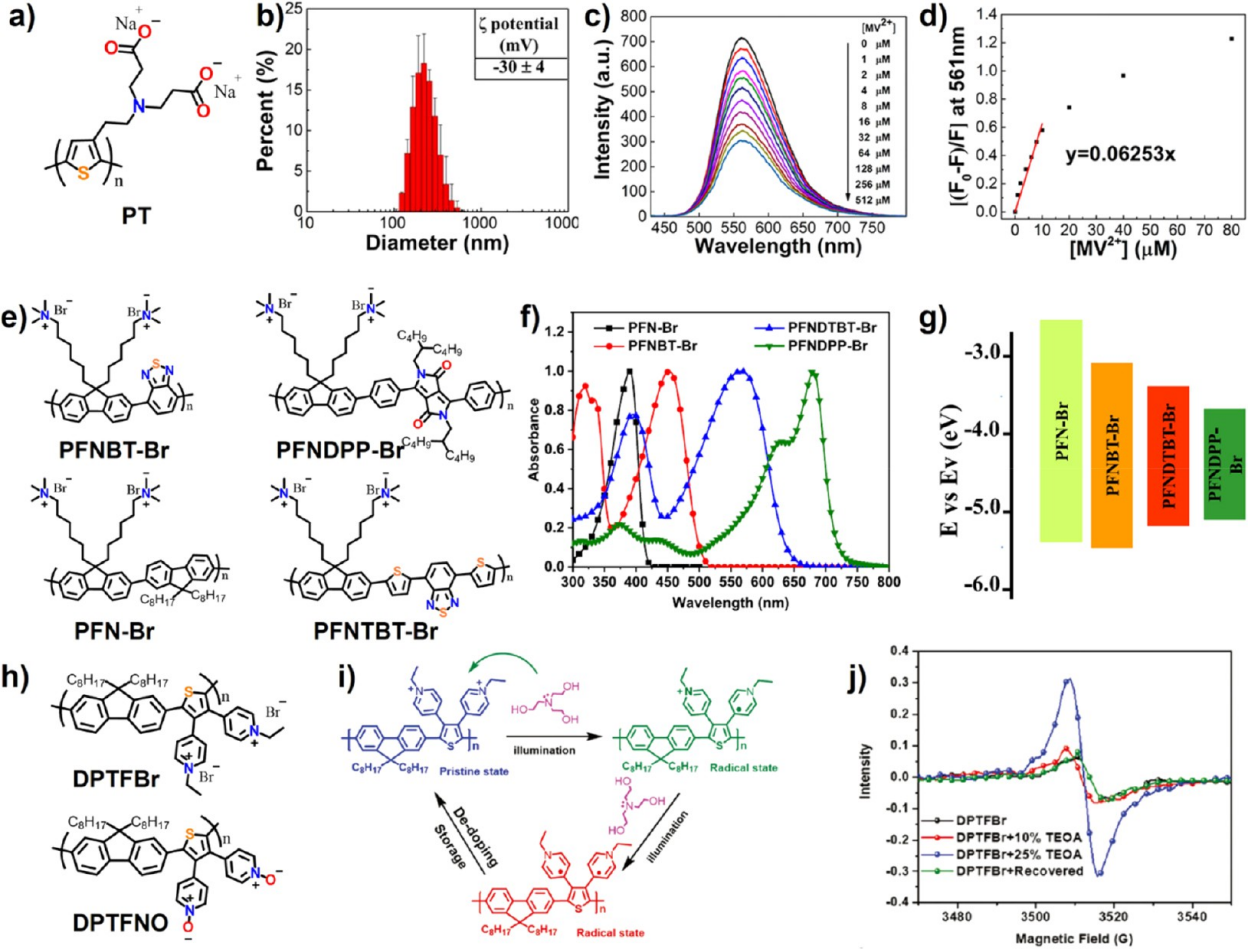
(a) Structure of sodium carboxylate-modified PT. (b) Diameter distribution and zeta potentials of PT aqueous solution. (c) Fluorescence intensity of PT with different contents of MV^2+^. (d) Stern–Volmer curve of quenching PT to MV^2+^. Reproduced with permission from ref 45. Copyright 2017 American Chemical Society. (e) Structure of PFN-Br, PFNDPP-Br, PFNBT-Br, and PFNDTBT-Br. (f) UV–vis spectra and (g) energy levels of cationic CPEs. Reproduced with permission from ref 47. Copyright 2019 Elsevier B.V. (h) Structure of DPTFBr and DPTFNO. (i) Photoinduced amine doping processes to DPTFBr. (j) ESR spectra of DPTFBr with different contents of TEOA. Reproduced with permission from ref 51. Copyright 2021 Royal Society of Chemistry.

The side chain of hydrophilic groups can act as a stabilizer for colloidal CPNs in water. To improve the stability of the colloidal system, Zhang’s group reported a new kind of conjugated poly­(benzothiadiazolylfluorene) (P-BT-Vim) bearing vinyl imidazolium electrolytes as the side chain, which could form the cross-linked CPs (P-FL-BT-3) in aqueous dispersion under irradiation with a white LED lamp (Figure 2a).^52^ At a low concentration of 0.10 mg mL^–1^, P-FL-BT-3 showed spherical nanoparticles (NPs) with a diameter of 85 nm (Figure 2b), resulting in its good dispersity in water. At a high concentration, a cross-linked P-FL-BT-3 hydrogel with interconnected pores of ∼50 mm was synthesized by self-initiation photopolymerization (Figure 2c). Such a hydrogel-like conjugated network would swell and generate exposed catalytic active sites in water and facilitate the stability of catalysts and products in a poor solvent. Additionally, a series of hydrogel photocatalysts of P-BT-G*X* (*X* = 0, 9, 19, and 41) were prepared by the photopolymerization of vinyl imidazolium-functionalized (P-BT-Vim) in the presence of anionic poly­(acrylic acid) (PAA).^53^ The cross-linked P-BT-G41 exhibited good deionized water absorption up to 470 times its weight. To achieve the efficient recycling use, the inorganic Fe_3_O_4_ NPs were embedded in the hydrogel to construct organic/inorganic hybrid photocatalysts of MCPs (Figure 2d). As shown in Figure 2e, such photocatalysts could be recollected from the solution using a magnet. Moreover, the wetting property of the hydrogel network can be controlled by changing the type of counteranions, expanding their dispersion both in water and organic solvent (Figure 2f).^54^

**Figure 2:**
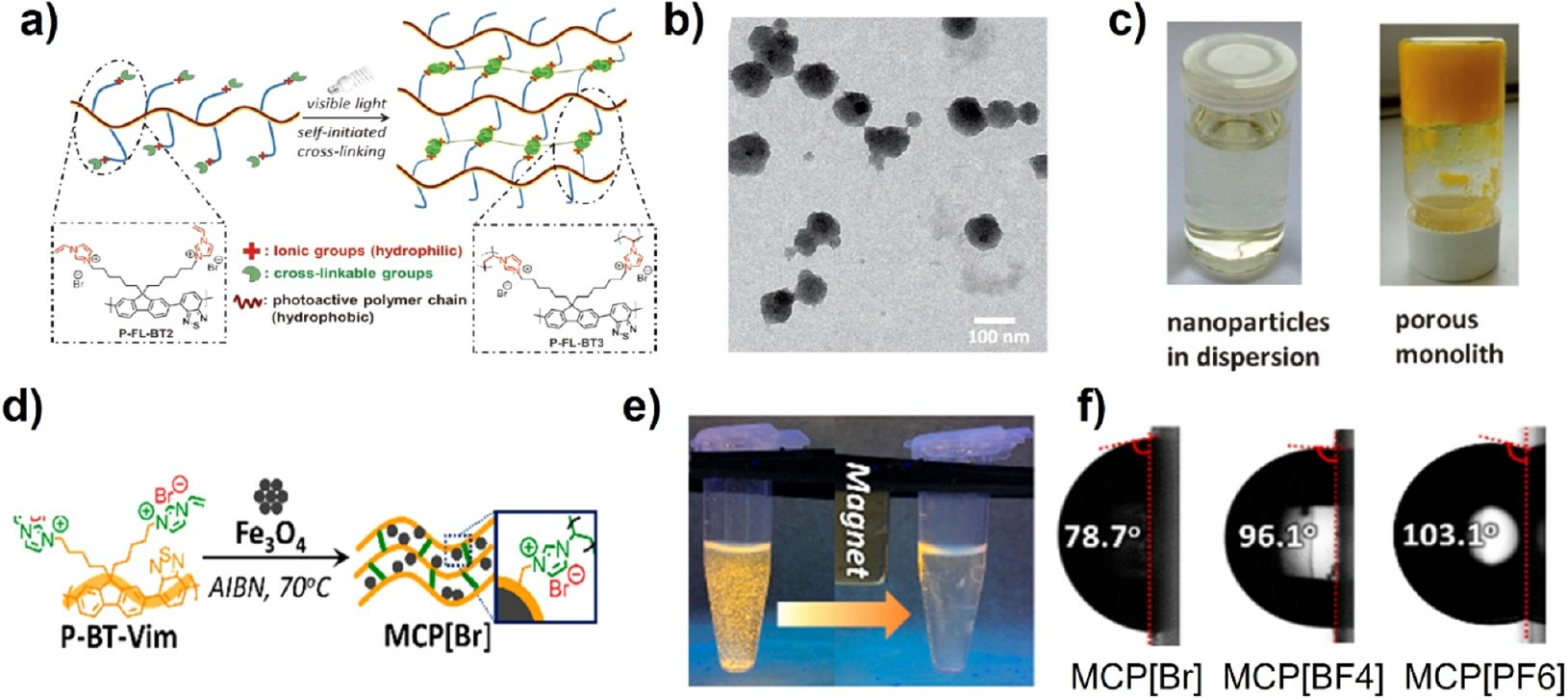
(a) Schematic synthesis of cross-linked P-FL-BT-3. (b) Transmission electron microscopy (TEM) image of P-FL-BT-3 NPs. (c) Photo of P-FL-BT-3 in water and porous monolith. Reproduced with permission from ref 52. Copyright 2015 Wiley-VCH. (d) Schematic preparation of MCP­[Br]. (e) Photograph of magnetic responsive MCP­[Br]. (f) Contact angle value of MCP­[Br], MCP­[BF_4_], and MCP­[PF_6_]. Reproduced with permission from ref 54. Copyright 2020 American Chemical Society.

The carboxylic acid and amine derivatives are typical organic acids and base, respectively. Thus, the side chain functionalization of CPNs with carboxylic acid or amine can effectively improve the hydrophilicity of the polymeric materials. In 2013, Vilela et al. constructed conjugated microporous poly­(benzothiadiazole) (CMP) by palladium (Pd)-catalyzed condensation of 4,7-dibromo BT with 1,3,5-triethynylbenzene, which was carboxylic acid-functionalized to prepare WCMPs (WCMP_0.1 and WCMP_0.4) via the thiol-yne click reaction with 3-mercaptopropionic acid (Figure 3a).^55^ These WCMPs showed a better aqueous dispersion with a concentration up to 1 mg mL^–1^ than that of unmodified CMP (Figure 3b). Using the same click reaction, different types of amine groups could be grafted on the conjugated microporous polymers to change their water contact angles.^56^ Compared with organic salt, the structure of the amine group has a great influence on the pH value of water, resulting in the stimulus-responsive CPs in aqueous solution. Zhang et al. found a tertiary amine-modified fluorine-based CP (P-BT-DEA), which could form the cationic P-BT-DEA-CO_2_ under the CO_2_ atmosphere (Figure 3c).^57^ As shown in Figure 3d, P-BT-DEA-CO_2_ exhibited good hydrophilicity in water and could be recovered to hydrophobic P-BT-DEA after bubbling with N_2_ gas. The reversible zeta potentials and pH values of polymeric aqueous solution could be achieved under treatment with CO_2_/N_2_ gas (Figure 3e).

**Figure 3:**
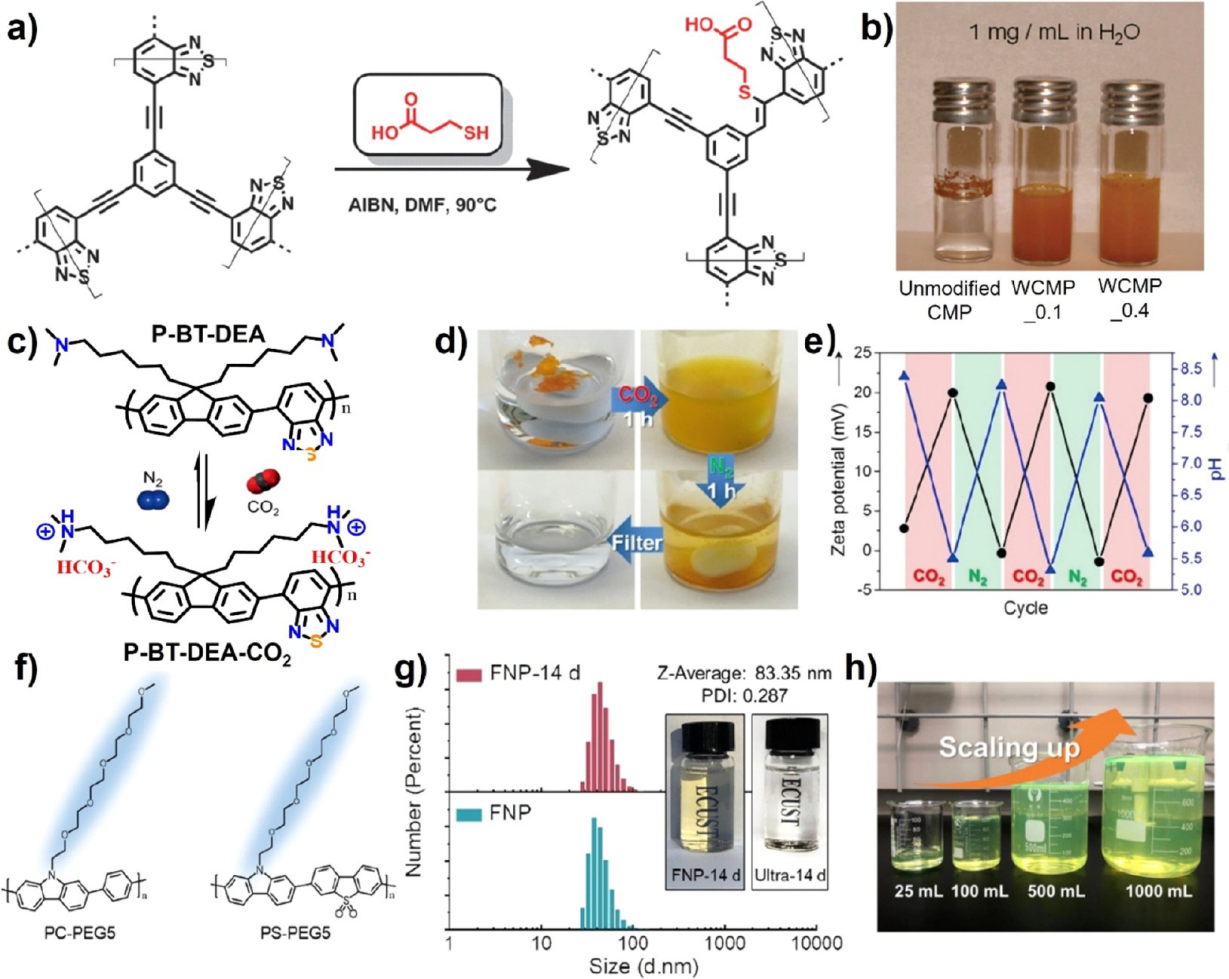
(a) Schematic synthesis of carboxylic acid-functionalized WCMPs and (b) photograph of unmodified CMP and WCMPs in water. Reproduced with permission from ref 55. Copyright 2013 Royal Society of Chemistry. (c) Schematic structure of CO_2_ responsiveness of P-BT-DEA and (d) photographs of P-BT-DEA in water after bubbling with CO_2_ and N_2_ gas. (e) Zeta potentials and pH values of P-BT-DEA upon reversible CO_2_/N_2_ treatment. Reproduced with permission from ref 57. Copyright 2018 Wiley-VCH. (f) Structure of PC-PEG5 and PS-PEG5; (g) DLS and photograph of PC-PEG5 NPs for 15 days; and (h) photograph of the PS-PEG5 NP with volume up to 1000 mL at the concentration of 1 mg mL^–1^ prepared by flash nanoprecipitation (FNP). Reproduced with permission from ref 61. Copyright 2021 Wiley-VCH.

Poly­(ethylene oxide) (PEO) is a kind of nonionic water-soluble polymer exhibiting unique properties like structural stability and biocompatibility, which has been applied in material, energy, and biology fields.^58^ As the fragment of PEO, oligo­(ethylene glycol) (OEG) is often introduced to modify the conjugated building blocks, such as benzodithiophene,^59^ pyrrolopyrroledione,^60^ carbazole,^61^ and benzothiadiazole,^62^ to improve the water dispersibility of CPs. Zhu and co-workers reported the synthesis of polycarbazole-based copolymers (PC-PEG5 and PS-PEG5) with PEO as the side chain (Figure 3f). Using the FNP technique, the homogeneous and stable colloidal NPs form with uniform size from tens-to-hundred nanometers. Taking the example of PC-PEG5, these NPs with an average diameter of 83 nm exhibited high size stability for 15 days (Figure 3g). This technique has been applied to produce polymeric NPs solution in a large scale to 1000 mL at the concentration of 1 mg mL^–1^ (Figure 3h).^61^

#### 2.1.2 End-Group Covalent Functionalization

Nanomaterials with controlled morphologies and different dimensions have been constructed from the CP-based block copolymers.^33^ Owing to the hydrophobic nature of CPs, the amphiphilic conjugated block copolymers could be prepared effectively by functionalizing the terminal group of CPs. In 2022, Tian and co-workers reported an amphiphilic triblock copolymer by grafting two poly­(*N*,*N*-dimethylamino ethyl methacrylate) segments on the end of polyfluorene as the acceptor (P2) and hydrophobic poly­(9,9-dioctylfluorene-*alt*-bithiophene) as the donor (P1) (Figure 4a).^63^ Owing to the matched energy levels of P1 and P2, the photoinduced energy transfer could be achieved in the P1/P2 binary heterojunction NPs (Figure 4b). This polymeric NP was mixed efficiently with HydA1 [FeFe]-hydrogenase via electrostatic interaction. The ns-transient spectroscopy studies demonstrated the efficient decay of MV^•+^ population in the presence of hydrogenase enzyme because of the electron transfer from the polymeric NP to the enzyme catalyst (Figure 4c).

**Figure 4:**
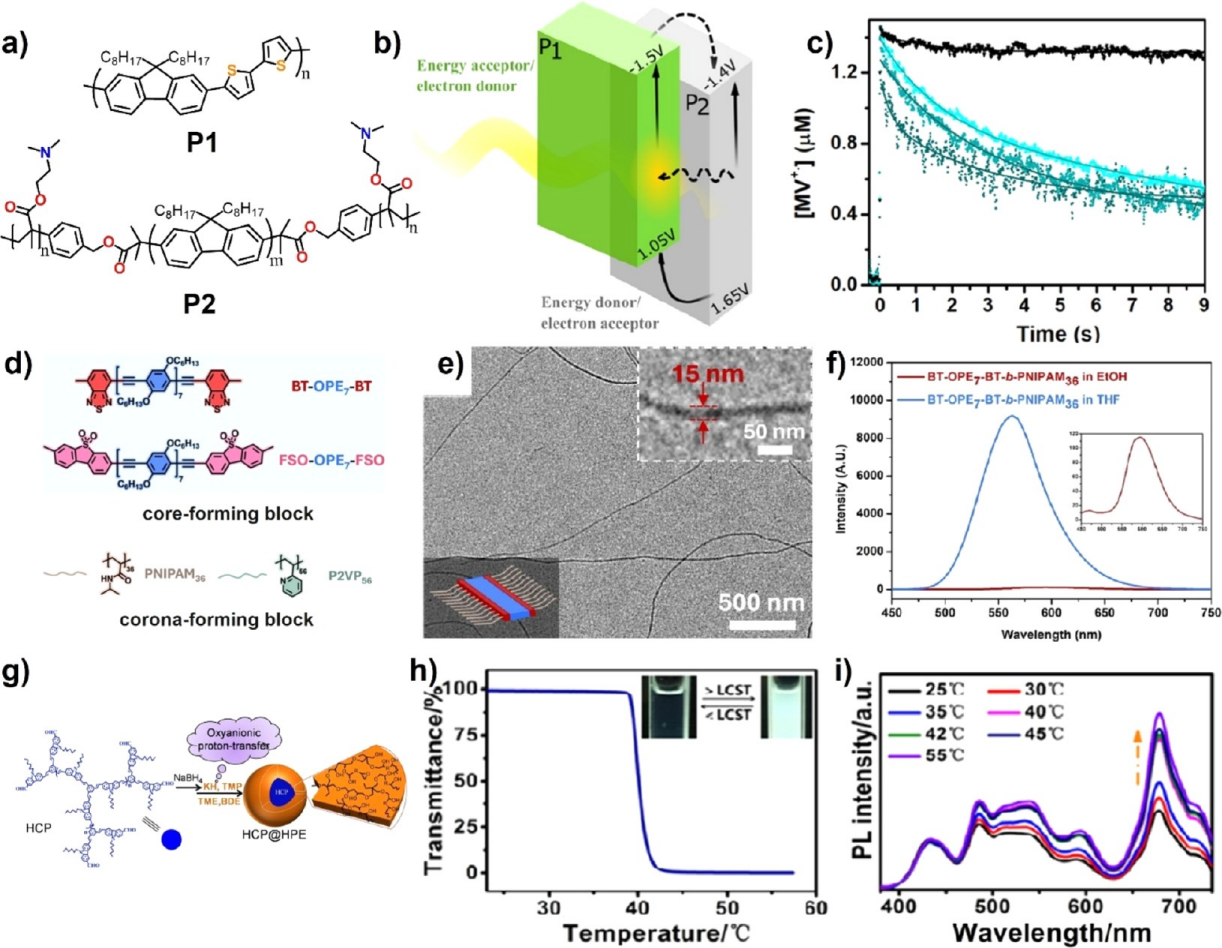
(a) Structures of P1 and P2. (b) Energy levels of P1 and P2 in the heterojunction NP. (c) TA kinetic of MV^•+^ at 603 nm for reaction mixtures with (cyan blue curve) and without hydrogenase (black curve). Reproduced with permission from ref 63. Copyright 2022 American Chemical Society. (d) The structures of BT-OPE_7_-BT, FSO-OPE_7_-FSO, PNIPAM_36_, and P2VP_56_. (e) TEM image of fiber-like micelles. (f) Photoluminescence (PL) spectra of BT-OPE_7_-BT-*b*-PNIPAM_36_ in tetrahydrofuran (THF) and ethanol. Reproduced with permission from ref 65. Copyright 2024 American Chemical Society. (g) Schematic synthesis route to HCP@HPE. (h) Temperature dependence of optical transmittance for HCP@HPE in an aqueous solution. (i) Thermal responsive PL spectra of the HCP@HPE-Ce6 aqueous solution. Reproduced with permission from ref 67. Copyright 2016 American Chemical Society.

Rigid π-conjugated polymers are facile to crystallize in the selected solvent. Thus, CP-based 1D nanofibers with controlled length/composition have been constructed from diblock copolymers containing crystalline CP as the core via living crystallization-driven self-assembly (CDSA).^64^ Recently, Feng and co-workers prepared two kinds of D–A-type oligo­(p-phenyleneethynylene)-based CPs (BT-OPE_7_-BT and FSO-OPE_7_-FSO) with a terminal alkyne, which was used as core-forming blocks. Poly­(2-vinylpyridine) (P2VP) or poly­(*N*-isopropylacrylamide) (PNIPAM) was prepared as corona-forming blocks (Figure 4d).^65^ Four kinds of diblock copolymers (BT-OPE_7_-BT-*b*-PNIPAM_36_, BT-OPE_7_-BT-*b*-P2VP_56_, FSO-OPE_7_-FSO-*b*-PNIPAM_36_, and FSO-OPE_7_-FSO-*b*-P2VP_56_) were constructed via click reaction. For BT-OPE_7_-BT-*b*-PNIPAM_36_, the BT-OPE_7_-BT adopted a face-to-face stacking motif, resulting in 1D fiber-like micelles in ethanol via both self-seeding and seeded growth CDSA approaches (Figure 4e). Compared with BT-OPE_7_-BT-*b*-PNIPAM_36_ in THF, the 1D micelles exhibited an obvious red-shift in both UV–vis and PL spectra with low fluorescence intensity due to the efficient π–π interaction of BT-OPE_7_-BT for exciton migration (Figure 4f).

Hyperbranched CP is the highly branched macromolecular architecture, having the specific advantage of compact structure, low-entanglement, and many functional terminal groups.^66^ Qiu et al. reported a conjugated unimicelle (HCP@HPE) by grafting of hyperbranched PEO (HPE) on the surface of hyperbranched CPs by the ring-opening polymerization method (Figure 4g).^67^ Such conjugated unimicelles have good luminescent properties in aqueous solution because the intermolecular interaction of the CP core is restricted efficiently by the PEO shell based on the “multimicelle aggregate” self-assembled mechanism, while HPE had a typical thermal-responsive lower critical solution temperature character, and the HCP@HPE exhibited the reversible transition of transparent and opaque solution in water (Figure 4h). After anchoring the acceptor of Chlorin e6 (Ce6) on the surface of HPE, the fluorescence resonance energy transfer from unimicelle to Ce6 was switched-on efficiently under NIR light irradiation with an accompanying photothermal effect (Figure 4i).

#### 2.1.3 Main-Chain Covalent Functionalization

Previous studies demonstrate that the extended π-conjugation of CPNs is necessary to broaden the optical absorption of polymeric photocatalysts for achieving a better catalytic performance. However, the ideal effective conjugation length for photocatalysis is still unclear. Furthermore, side-chain-engineered CPNs still exhibit the electron–hole recombination in the aggregate state, which needs a similar issue in conventional CPNs.^68^ Chou et al. developed a novel main-chain engineering approach to prepare a random copolymer containing poly­(fluorenyl-*co*-phenylbenzo­[*b*]­phosphindole) and OEG with different lengths (Figure 5a).^69^ After incorporation of hydrophilic nonconjugated segments in the backbone of CPs, their optical properties, energy levels, and exciton lifetimes showed no obvious difference. However, when these polymers were coated on the silicon wafers, the P-HEG-10 film showed a lower water contact angle (65.5°) than that of hydrophobic poly­[(9,9-dioctyl-9H-fluorenyl-2,7-diyl)-*co*-(5-phenylbenzo­[*b*]­phosphindole-5-oxide-2,7-diyl)] (PFBPO) (79.3°) after dropping for 20 min due to the existence of hydrophilic OER segments (Figure 5b). To further confirm this, the average hydrogen bond number and radial distribution function of these CPNs containing nonconjugated segments were evaluated by molecular dynamics simulation. Theoretical calculations revealed that P-HEG-10 with 10 mol % HEG-modified showed the highest possibility of hydrogen bonds with water (Figure 5c,d). Huang and co-workers reported a new kind of pyridinium-based hyperbranched CPEs by nucleophilic substitution reaction of 1,3,5-trispyridylbenzene with different bromo-containing conjugated units (e.g., pyrrolopyrroledione, perylenediimide, and naphthalenediimide).^70^ Owing to the high ratio of the pyridinium group in the main chain of CPEs, these polymers were highly soluble in various polar solvents, such as dimethyl sulfoxide, dimethylformamide, methanol, and acetonitrile.

**Figure 5:**
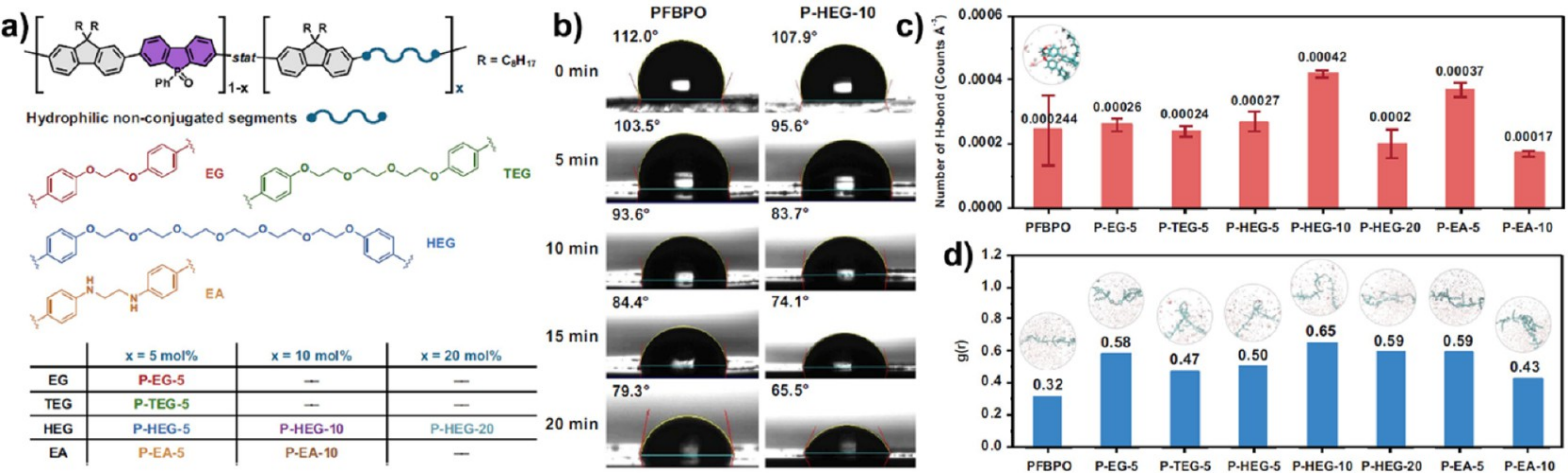
(a) Structure of main-chain engineering of CPs. (b) Contact angles of PFBPO and P-HEG-10. (c) The statistics of hydrogen bonds and (d) hydrogen bond formation of main-chain engineering of CPs via molecular dynamics study. Reproduced with permission from ref 69. Copyright 2022 Springer Nature.

### 2.2 Supramolecular Chemical Strategy

#### 2.2.1 Small Molecular Surfactants

Molecular self-assembly is a spontaneous tool for the ordered organization of molecular building blocks through noncovalent supramolecular interaction.^28^ Nanoprecipitation technique developed by Fessi et al. has been used in the pharmaceutical, biological, and agricultural research areas, which has the advantage of scale-up production, good reproducibility, and uniform size distribution.^71^ Small molecular surfactants consisting of hydrophilic and hydrophobic groups can reduce the interfacial tension of CPs in polar solvents.

Sodium dodecyl sulfate (SDS) is a classical commercial surfactant. Chen et al. reported the preparation of conducting P3HT NPs by nanoprecipitation technique with SDS as a stabilizer, which can be used as light-harvesting NPs to enhance the conversion efficiency and selectivity of graphene oxide (iGO) as the catalysts via improving the interfacial charge transfer.^72^ Organic heterojunctions constructed with a donor and an acceptor can extend the optical absorption band and drive exciton dissociation for spatial charge separation. McCulloch and co-workers reported to prepare the D/A heterojunction using poly­(benzo­[1,2-*b*;3,3-*b*]­dithiophene]-thieno­[3,4-*b*]­thiophene) derivative (PTB7-Th) as the donor and nonfullerene EH-IDTBR as the acceptor (Figure 6a).^27^ TEM images showed that SDS with a long aliphatic tail showed a higher affinity to PTB7-Th than that of EH-IDTBR, resulting in the formation of core–shell NPs with EH-IDTBR core and PTB7-Th shell structure (Figure 6b). However, the D/A heterojunction of PTB7-Th/EH-IDTBR NPs can be readily prepared by using 2-(3-thienyl)­ethyloxybutylsulfonate sodium salt (TEBS) with a short aromatic tail as the stabilizer, resulting in high external quantum efficiencies (EQEs: >5%) and improved charge extraction to the interface of NPs/cocatalyst. These polycrystalline structures were further confirmed by small-angle neutron scattering (SANS) analysis, demonstrating that NPs formed with TEBS showed a more intermixed D/A heterojunction than that with SDS (Figure 6c). Recently, Kuila constructed the positive organic NPs by blending P3HT with oleylamine as the electron donor core, which can form the core–shell-type NPs (P3HT–PCBA) with a size of ∼200 nm using negative phenyl C-61 butyric acid (PCBA) as the electron acceptor shell by an electrostatic interaction (Figure 6d).^73^ The DLS result showed that the P3HT-PCBA NPs had an average diameter of 256 nm with broad size distribution (Figure 6e). After hybridization with PCBA, the PL lifetime of P3HT-PCBA decreased to 368 ps (Figure 6f), suggesting an efficient electron transfer process between P3HT and PCBA.

**Figure 6:**
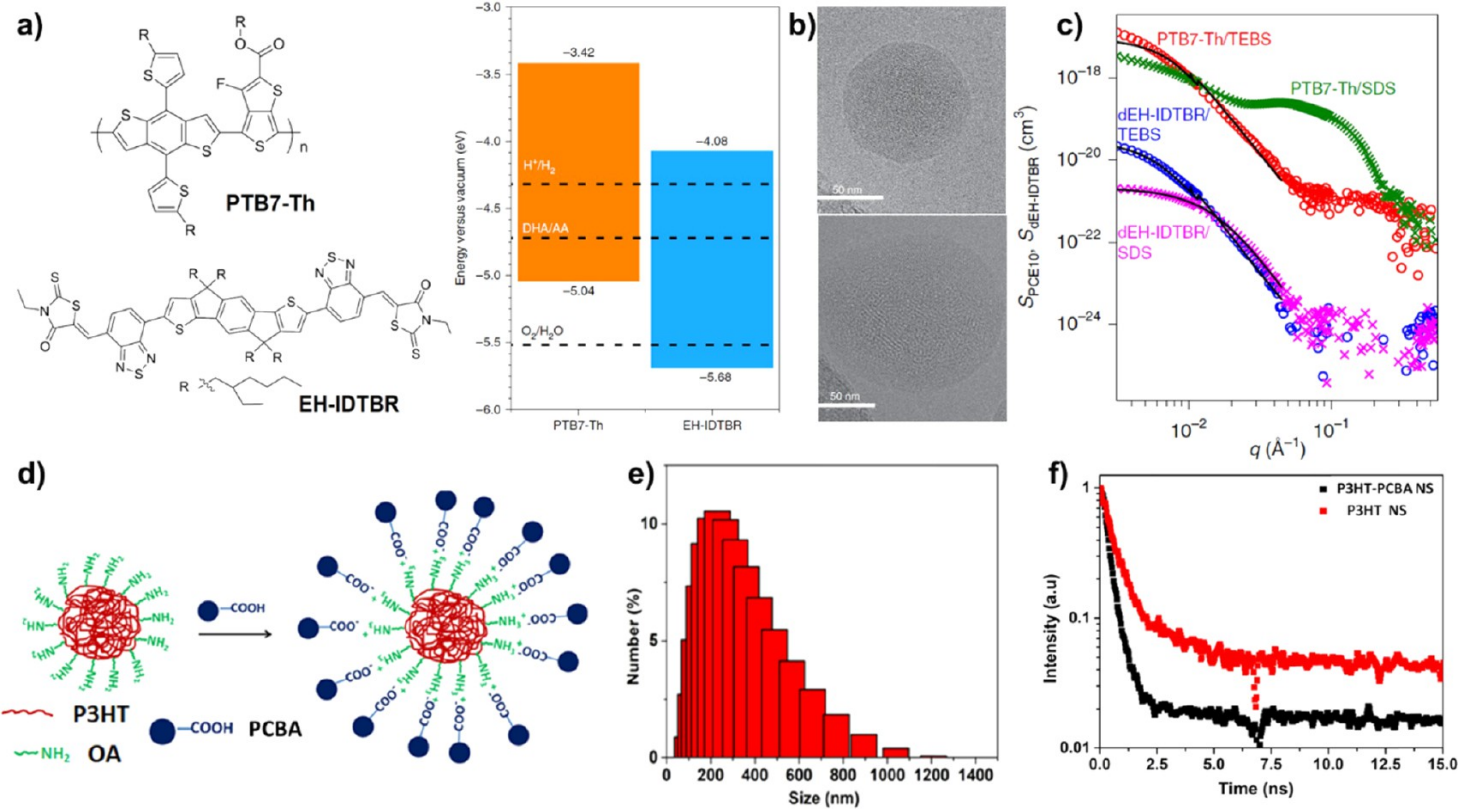
(a) Structures and energy levels of PTB7-Th and EH-IDTBR. (b) TEM images of the heterojunction of PTB7-Th/EH-IDTBR NPs by using SDS (top) and TEBS (bottom) as surfactants. (c) SANS of various heterojunctions in H_2_O/D_2_O solutions. Reproduced with permission from ref 27. Copyright 2020 Springer Nature. (d) Schematic synthesis route to P3HT-PCBA NPs; (e) Size and distribution of P3HT-PCBA NPs. (f) Time-resolved emission decay curves. Reproduced with permission from ref 73. Copyright 2022 Royal Society of Chemistry.

#### 2.2.2 Amphiphilic Diblock Copolymers

Diblock copolymers consisting of a distinct hydrophilic polymer segment and a hydrophobic polymer segment covalently linked at the chain end can self-assemble into the ordered nanostructures with typical morphologies (e.g., spherical, rod, and vesicle) and nanometer size of 10–100 nm in a selective solvent.^74^ Tian et al. first reported the novel organic photocatalytic NPs using the diblock copolymer as the stabilizer.^75^ In the mixture of THF and water, poly­(fluorenyl-*co*-benzothiadiazole) (PFBT) was wrapped into the interior of micelle of the amphiphilic polystyrene-based copolymer (PS-PEG-COOH) (Figure 7a). After THF was removed, the PFBT would homogeneously disperse in water. TEM and DLS results revealed the formation of NPs with an average size of 40 nm (Figure 7b). The red-shift of 20 nm in both UV–vis and PL spectra suggested the existence of *J*-type π–π interaction of PFBT in an aqueous solution (Figure 7c). To improve the absorption of sunlight, the absorption band of NPs could be facilely controlled by changing the intramolecular charge transfer in the backbone of D–A-type CPs.^76^ The noble-metal-chelating organic complexes show specific optical absorption and catalytic activity. Chou’s group developed a series of platinum (Pt) or iridium (Ir)-based donor–acceptor-metal CPs, which self-assembled into NPs under the emulsification of PS-PEG-COOH.^77–79^ In addition, the binary and ternary heterojunction organic NPs were also prepared in the PS-PEG-COOH aqueous solution by Chou’s and Tian’s group, respectively.^80–82^ Under high ultrasonication, PS-PEG-COOH-stabilized PFODTBT formed the well-dispersed droplets, which could aggregate into a hollow nanostructure with a porous polymer shell.^83^

**Figure 7:**
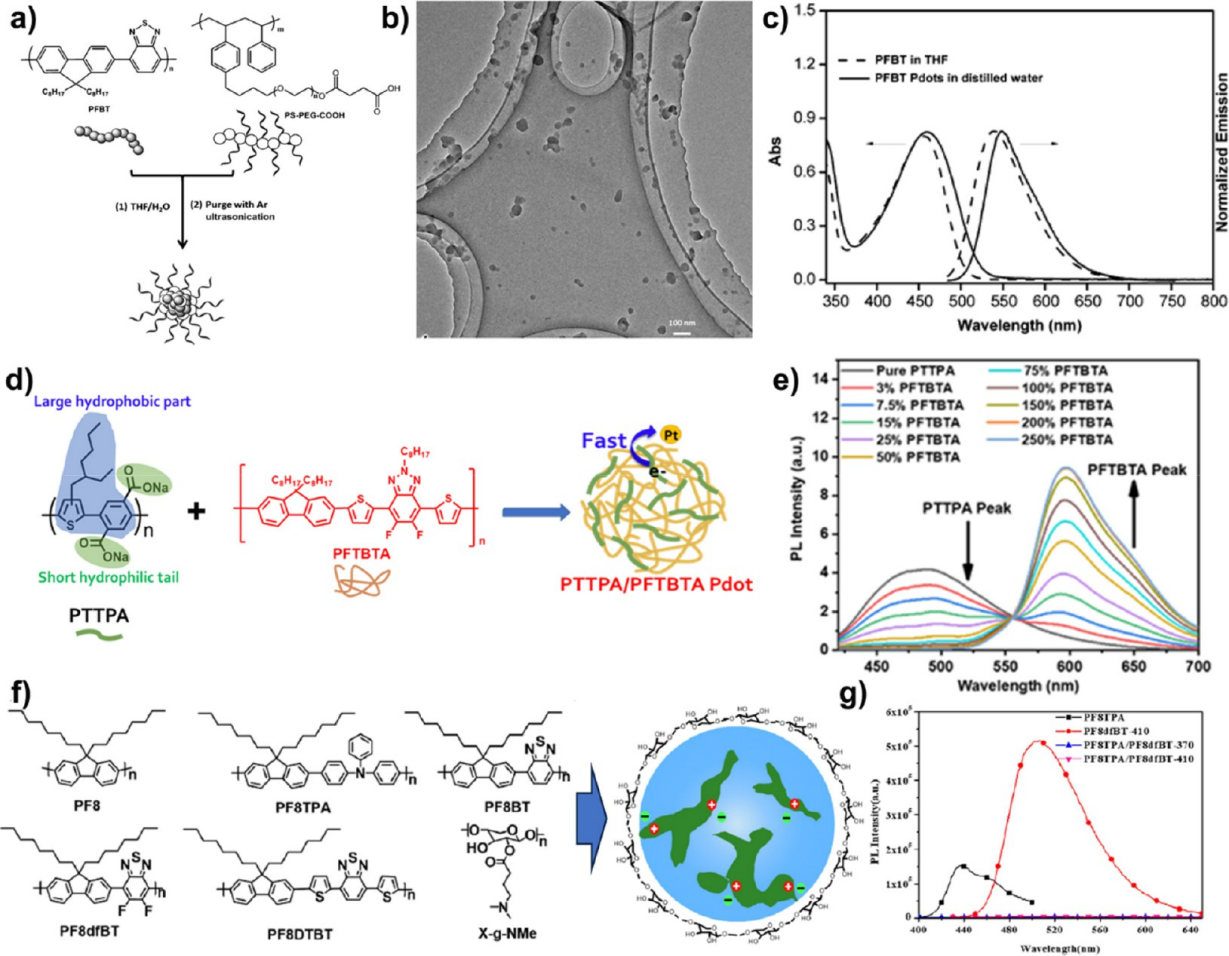
(a) Schematic illustration of PFBT NPs using PS-PEG-COOH as a stabilizer. (b) TEM image of PFBT NPs. (c) UV–vis and PL spectra of PFBT in THF and water. Reproduced with permission from ref 75. Copyright 2016 Wiley-VCH. (d) Preparation of PTTPA/PFTBTA NPs. (e) PL spectra of PTTPA/PFTBTA NPs with different contents of PFTBTA. Reproduced with permission from ref 88. Copyright 2021 American Chemical Society. (f) Schematic illustration of CP NPs using X-*g*-NMe as a stabilizer. (g) PL spectra of PF8TPA, PF8dfBT, and their blends. Reproduced with permission from ref 91. Copyright 2019 American Chemical Society.

Apart from PS-PEG-COOH, other amphiphilic diblock copolymers (poly­(ethylene glycol-*b*-methyl methacrylate)) (PEG-*b*-PMMA),^84^ poly­(3-hydroxybutyrate) (PHB),^85^ and poly­(styrene-*co*-maleic anhydride) (PSMA)^86,87^ are reported as the macromolecular surfactants for the preparation of photocatalytic conjugated micelles. However, such nonconjugated amphiphilic polymers have a negative effect on the photocatalytic efficiency of the conjugated polymeric micelles due to the suppression of the electron transfer between the CP and Pt cocatalyst. Chou et al. used the amphiphilic CPE (PTTPA or PBTTPA) as the surfactant to stabilize the hydrophobic PFTBTA in water for construction of the binary heterojunction polymer photocatalysts (Figure 7d).^88^ Compared with PS-PEG-COOH, the water contact angle of PTTPA/PFTBTA NPs was higher than that of the pure PFTBTA, suggesting that the CPEs are randomly distributed with PFTBTA for efficient intracellular electron transfer. Thus, the PL spectra of PTTPA/PFTBTA NPs could be controlled by changing the ratio of PFTBTA in these NPs (Figure 7e).

#### 2.2.3 Biomass Materials

Polysaccharides are one of the most important biomass materials in nature. The amphiphilicity of these biomacromolecular materials can be obtained by structural modification.^89^ Owing to the high biosafety, biomass materials have been applied as surfactants in the field of biomedical materials, including enzyme immobilization, hydrogel, nucleic acid delivery, etc.^90^ Huang and co-workers used Xylan derivative (X-*g*-NMe) to wrap D–A-type CPs with different energy levels to form the NPs (Figure 7f).^91^ The heterojunction structure in these NPs was also easy to achieve by blending two CPs. PL spectra results indicated the efficient fluorescence quenching in the system of PF8TPA/PF8BT and PF8TPA/PF8dfBT, suggesting the presence of a strong FRET process (Figure 7g). In addition, amphiphilic glucan microbeads of Sephadex LH-20 was reported as a supporter to immobilize the CP.^92^ The fluorescence microscopy images demonstrated that the CP was dispersed homogeneously on the hybrid microbeads.

### 2.3 In Situ Polymerization Strategy

#### 2.3.1 Soft-Template Nanoreactors

The soft-template method is a simpler and more efficient way for the preparation of CPNs, which usually requires a mild polymerization condition. Chemical oxidative polymerization is exploited for the synthesis of the CPs from aromatic monomers (e.g., aniline, pyrrole, thiophene, etc.) under strong oxidative agents, like ammonium persulfate or iron­(III) chloride. Owing to the fast polymerization process and mild reaction condition, the oxidation polymerization has been applied for the synthesis of the CPs in the confinement environment.^93^ In 2012, Wang and co-workers prepared PT NPs by microemulsion polymerization of thiophene in the presence of nonionic surfactant Triton X-100.^94^ Under a similar oxidation polymerization condition, 3-hexylthiophene and pyrrole can also be in situ polymerized into the photocatalytic NPs with cationic (cetyltrimethylammonium bromide (CTAB)) or anionic SDS surfactants.^95,96^ The Pd–catalytic cross-coupling reaction is an efficient synthetic method for the preparation of the CP. In 2019, Cooper et al. developed a one-pot polymerization of porous conjugated polymeric nanodots for the photocatalytic application (Figure 8a).^97^ The reactants and Pd catalyst were formed by the miniemulsions with SDS in the mixture of toluene and water. After heating overnight at 90 °C, the resulting NPs showed the high dispersity in water, and no aggregation phenomenon was found over 11 days without stirring (Figure 8b). As the example of P10-e, the TEM image revealed that the hydrodynamic diameter of NPs was around 150 nm (Figure 8c).

**Figure 8:**
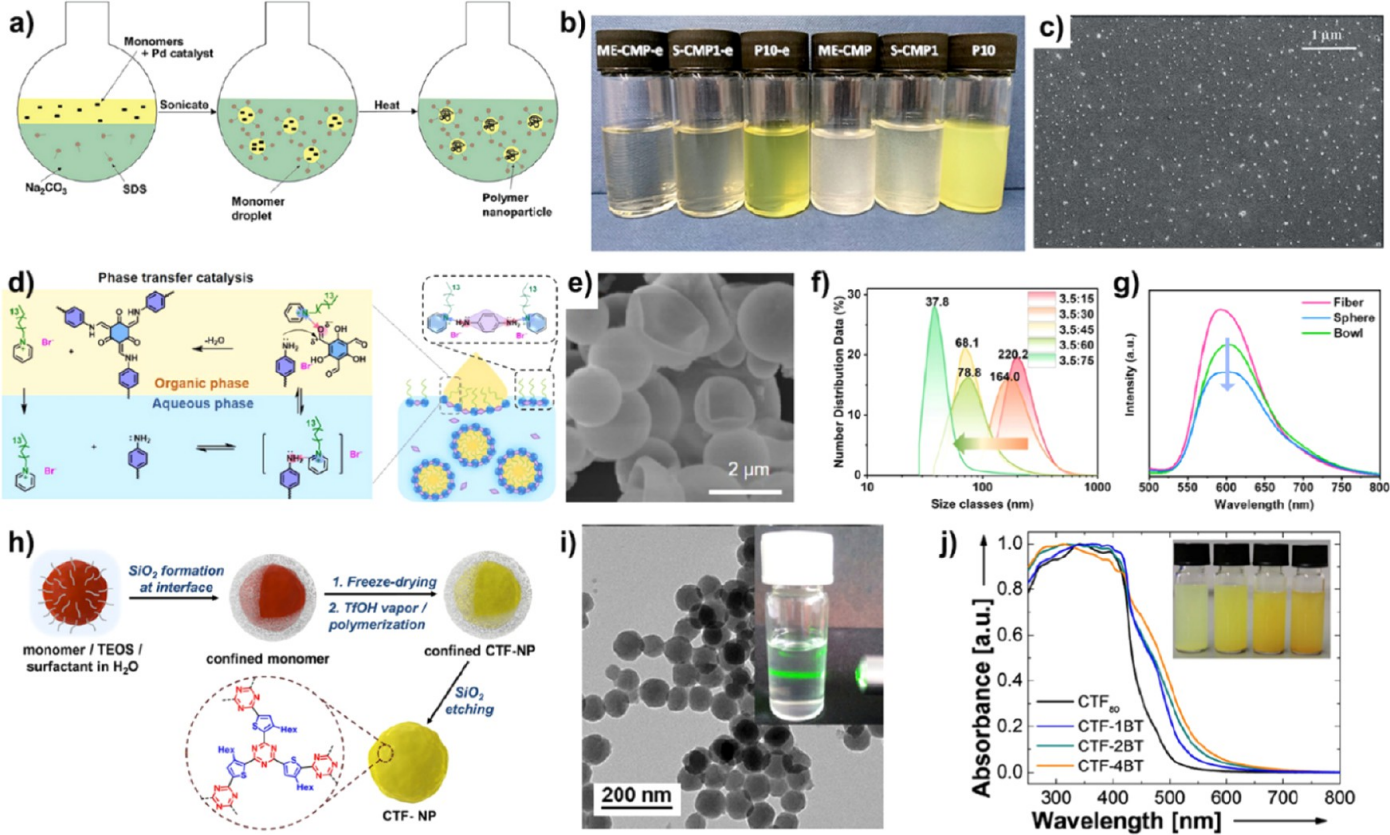
(a) Schematic illustration of the CP using the Pd-catalytic cross-coupling reaction. (b) Photographs of CP microemulsion in water. (c) Scanning electron microscopy (SEM) image of P10-e. Reproduced with permission from ref 97. Copyright 2019 Royal Society of Chemistry. (d) Schematic illustration of TpPa-COF via microemulsion polymerization. (e) SEM image of bowl shape of TpPa-COF. (f) Controlled size of TpPa-COF prepared by the varying ratio of oil/water. (g) PL intensity of TpPa-COF with varying shapes. Reproduced with permission from ref 100. Copyright 2023 American Chemical Society. (h) Schematic synthesis route to covalent triazine framework (CTF) NPs using silica as the hard template. (i) TEM image of CTF NPs. (j) UV–vis spectra of CTF NPs with varying BT content. Reproduced with permission from ref 103. Copyright 2020 Wiley-VCH.

COFs are a kind of porous organic polymer with ordered building block stacking, which could be synthesized by various reversible polycondensation reactions. Such a crystalline topological structure with a bicontinuous π-columnar array exhibited the fast charge separation and high exciton transfer, which has been confirmed as the promising candidate for photocatalytic applications.^98^ Traditional synthesis of COFs in an organic environment suffers from harsh conditions, which is unfavorable to the thermodynamic stability of the soft template. Zheng’s group reported that the imine-linked COFs could be constructed as the single crystal in the mild polymerization condition using amphiphilic amino-acid as the soft template.^99^ Recently, Jin and co-workers reported the preparation of ketoenamine-linked COFs (TpPa-COF) in the water–dichloromethane mixed solution by using pyridinium derivatives as the phase transfer catalyst (Figure 8d).^100^ The electrostatic interaction between the monomer of *p*-phenylenediamine and the pyridinium surfactants generated the micelles in H_2_O, which could activate the aldehyde group of 1,3,5-triformylphloroglucinol (Tp) for emulsion polymerization by the dipole interaction. TpPa-COF with different morphologies (e.g., spheres, bowls, and fibers) could be prepared via emulsion polymerization. The SEM image of Figure 8e showed the typical bowl NPs with an average size of 1.2 μm. The size of TpPa-COF could be realized by controlling the alterable emulsion conditions (Figure 8f). The morphology of TpPa-COF has a great influence on their fluorescence properties, suggesting that the charge migration and separation in the spherical TpPa-COF were faster than those in bowls and fibers (Figure 8g).

#### 2.3.2 Hard-Template Nanoreactors

Compared with the micelles from the organic materials, the inorganic materials possess a high structural stability when used as nanoreactors. Due to the presence of the large amount of hydroxy group on the surface of silica, it can be applied as nanoreactors in aqueous solution.^101^ Yang’s group prepared covalent organic polymer by the polycondensation reaction of 2,4,6-triformylphloroglucinol (TP) and 1,3,5-tris­(4-aminophenyl)­benzene (TAPB) in the emulsions of CTAB/SDS using acetic acid as the catalyst.^102^ Subsequently, the organic NPs were coated with silica via the hydrolysis of tetraethyl orthosilicate, which renders the rigid shell to restrict the aggregation of the polymer and improve their hydrophilic properties. Owing to the high acid resistance of silica, Zhang and co-workers prepared hybrid NPs by encapsulation of silica to cyan-based conjugated monomer micelles stabilized with cetyltrimethylammonium chloride in aqueous media.^103^ Then, the obtained particles were kept in the atmosphere of trifluoromethanesulfonic acid vapor for 24 h, and the corresponding CTF NPs were in situ polymerized inside the silica NPs, which was finally acquired by etching silica with NH_4_HF_2_ (Figure 8h). As-prepared NPs were suspended in diluted THF, showing the typical Tyndall effect. The TEM image revealed that these CTF NPs showed an average size of 80 nm with a uniform distribution (Figure 8i). The optical properties and energy level of CTF NPs can be tuned effectively by adjusting the content of the BT acceptor (Figure 8j).

## 3 Photocatalytic Applications of Conjugated Polymeric Nanomaterials

### 3.1 Organic Pollutant Degradation

Rapid industrialization is inseparable from water consumption. The generation of wastewater causes serious pollution to the environment and human health.^104^ Photocatalysis has been proven as a green and economical approach to bringing environmental pollution under control. Inorganic semiconductors (e.g., TiO_2_) exhibit the excellent photoredox properties for the degradation of organic pollution under the UV light. However, they have some intrinsic drawbacks, like toxicity of transition metal, photobleaching effect, and high cost, limiting their photocatalytic application in the industrial scale.^13^ Metal-free organic conjugated catalysts having tunable light-absorbing properties have been found to have broad utility in photovoltaics and chemical synthesis. To achieve their better recycling ability, the heterogeneous photocatalysts have gained great attention in the past decade.^105^ Scientists found that PT and polypyrrole (PPy) exhibited higher photodecomposition rate under visible-light irradiation than TiO_2_ due to negligible optical absorption above 400 nm.^95,96^ However, the photodecomposition efficiencies of PT and PPy did not meet the requirements of actual application demand. Kuila and co-workers reported that the P3HT-PCBA with a core–shell D–A-type structure showed the high photodecomposition efficiency with 82.5% of methylene blue (MB) degradation for 6 h visible-light irradiation using a 20 W white LED. Compared with that of P3HT-PCBA, the photodecomposition efficiency of pure P3HT was 34%.^73^ The experimental results indicated that the electron played a dominant role in the degradation of MB. Owing to the good electron acceptor nature of PCBA, the photoinduced exciton was easily dissociated in the D–A system of P3HT-PCBA.

To improve the photodecomposition efficiency against chemical pollution, Zhang’s group used the cross-linked P-FL-BT-3 NPs as the photocatalyst for the degradation of organic chemicals and heavy metal ions under a white LED lamp with 1.2 W cm^–2^ (Figure 9a).^52^ Under visible-light irradiation for 70 min, the photodecomposition efficiencies of MB and rhodamine B (RB) could exceed 90%, which was much higher than that of hydrophobic polyfluorene (Figure 9b). The photodegradation mechanism of P-FL-BT-3 against RB was evaluated by the addition of different scavengers. These results demonstrated that generation of superoxide (^•^O_2_^–^) was responsible for the degradation of the organic dye.^106–108^ It can also be used for the photoconversion of toxic Cr^VI^ ions into less harmful Cr^III^ after 120 min illumination (Figure 9c). P-FL-BT-3 had a conduction band at −1.13 eV in the excited state, which was higher than the potential for the reduction reaction of Cr^VI^ to Cr^III^. Moreover, the photodegradation performance of P-FL-BT-3 exhibited no significant decline after ten repeated photocatalytic cycles, suggesting the high stability and reusability of the P-FL-BT-3 material. The hydrogel photocatalysts of P-BT-GX from P-BT-Vim showed the high-water compatibility, resulting in the good distribution of photoactive sites (Figure 9d).^53^ Thus, P-BT-G41 exhibited the best photodecomposition efficiency of RB, which was degraded completely within 20 min under the white LED with a power of 0.07 W cm^–2^ and λ > 420 nm (Figure 9e). Such a swollen hydrogel could be assembled in the flow column to obtain a flow photoreactor setup, in which the RB could be efficiently degraded under visible-light irradiation for 3 min and removed from the column by flow washing with water (Figure 9f). Furthermore, this flow photoreactor setup with P-BT-G41 showed fewer photodegradation efficiency loss after several repeated cycles. Later, they investigated the photodegradation of tetracycline (TC) by using hybrid MCPs containing P-FL-BT-3 and Fe_2_O_3_ as a photosensitizer using a fluorescent light (24 W, λ > 400 nm).^54^ The type of the counterion played an important role in their photodecomposition efficiency. MCP­[Br] with good water compatibility showed the best radical generation, resulting in the highest degradation efficiency of TC in an aqueous solution than those of MCP­[BF_4_] and MCP­[PF_6_]. However, Fe_2_O_3_ NPs had a negative effect on the photodecomposition efficiency, restricting the light absorbance of the hybrid materials in the visible-light region.

**Figure 9:**
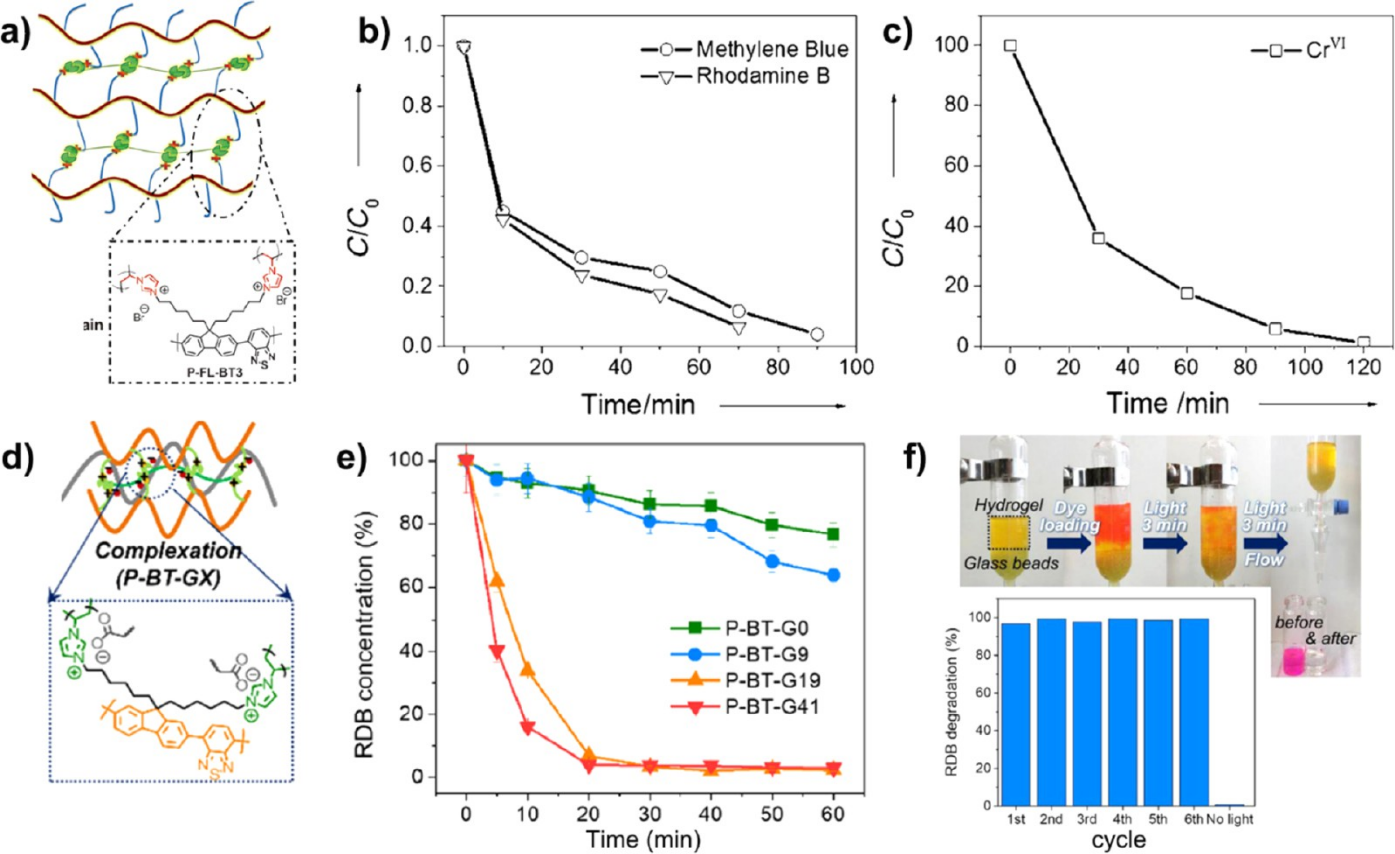
(a) Structure of cross-linked P-FL-BT-3. (b) Photocatalytic degradation of MB and RB using P-FL-BT-3 in water under visible-light irradiation. (c) Photocatalytic reduction of Cr ions using P-FL-BT-3 in water under visible-light irradiation. Reproduced with permission from ref 52. Copyright 2015 Wiley-VCH. (d) Structure of cross-linked P-BT-G*X* (*X* = 0, 9, 19, and 41). (e) Photocatalytic degradation of RB using P-BT-G*X* (*X* = 0, 9, 19, and 41) in water under visible-light irradiation. (f) Photographs of a flow column photoreactor with P-BT-G41 for photocatalytic degradation of RB and its cycle performance. Reproduced with permission from ref 53. Copyright 2019 American Chemical Society.

### 3.2 Chemical Transformations

Traditional chemical reactions are conducted to overcome the chemical barrier by inputting the external thermal energy, which suffers from some shortcomings, such as high energy consumption, low reaction rates, etc. In contrast, the photoinitiated redox reaction has been proved as a green and economically chemical process in polar solvents such as water and alcohols using NP as the heterogeneous photocatalysts. In photocatalytic reactions, photogenerated charge carriers (electrons and holes) interact with electron acceptors or donors on the material’s surface, producing highly active intermediate species, which can be applied to the conventional oxidation or reduction reactions.^20^

#### 3.2.1 Photooxidation Reaction

Vilela et al. reported the conversion of furoic acid to 5-hydroxy-2­(5H)-furanone in water using WCMPs as the photocatalyst.^55^ Under 420 nm-UV light irradiation with 12 W output in the O_2_ atmosphere, the modified WCMPs exhibited a higher reaction conversion than that of unmodified CMP due to the enhanced wettability of WCMPs. However, the chemical conversion was limited by the ^1^O_2_-induced degradation of WCMPs. Introduction of BT into the backbone of CPs would extend their light absorption band. Zhang and co-workers investigated the photooxidative coupling of benzylamine derivatives to imine-containing products using a white LED light illumination (20 W, λ > 420 nm) in the O_2_ atmosphere.^54^ The chemical conversion efficiencies of MCP­[BF_4_] and MCP­[PF_6_] are higher than that of MCP­[Br] due to their better dispersion of MCP­[BF_4_] and MCP­[PF_6_] in acetonitrile. Moreover, this photochemical reaction presented electron-withdrawing/donating substituent tolerance, achieving nearly 100% product conversion within 3 h illumination. Recently, Feng et al. carried out the photooxidation of sulfides to sulfoxide using the BT-OPE_7_-BT-*b*-PNIPAM_36_ nanofiber as the catalyst.^65^ Under an O_2_ atmosphere at room temperature, the desired sulfoxides with 99% conversion and >94% selectivity were realized for 3 h Xe-lamp illumination with a 300 W Xe lamp and a 420 nm cutoff filter. Both time-resolved fluorescence and electron paramagnetic resonance results demonstrated that ^1^O_2_ and ^•^O_2_^–^ were the crucial active intermediates for the photooxidation reaction. Using methanol as the solvent, the generation of H_2_O_2_ from these active intermediates can avoid the degradation of polymeric NPs but would further oxidize MeOH to obtain formate in the alkaline condition, which was confirmed by nuclear magnetic resonance spectra.^82^ Zhang’s group reported the photocatalytic oxidative [3+2] cycloaddition for the preparation of 2,3-dihydrobenzofuran derivatives using CTF NPs as the catalyst and ammonium peroxodisulfate as the terminal oxidant under a blue LED lamp (λ = 460 nm, 0.061 W cm^–2^) for 10 h (Figure 10a).^103^ These CTF NPs exhibited enhanced photocatalytic oxidative [3+2] cycloaddition performances compared to those of the bulk CTF material owing to their improved charge separation and delocalization (Figure 10b). Moreover, conversions after 10 h reaction revealed that both nanoscale size and electronic structure in the backbone of CTF have a great influence on the reaction conversion efficiency (Figure 10c).

**Figure 10:**
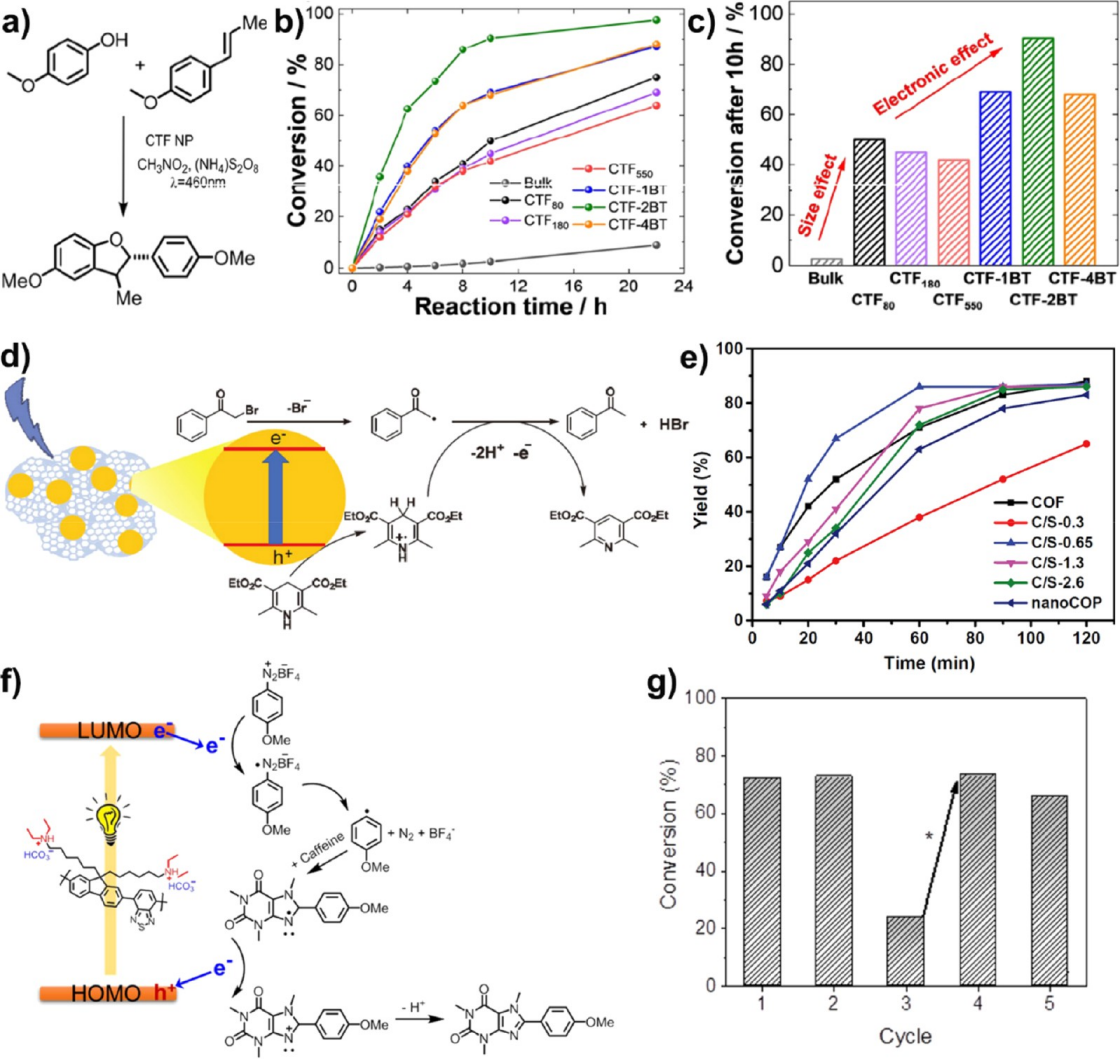
(a) Synthesis route to photooxidation [3+2] cycloaddition of 2,3-dihydrobenzofuran derivatives. (b) Photooxidation reaction kinetic using varying CTF NPs as the photocatalyst. (c) Conversion after 10 h reaction of different CTF NPs. Reproduced with permission from ref 103. Copyright 2020 Wiley-VCH. (d) Proposed photoreduction mechanism for the dehalogenation reaction. (e) Photocatalytic reaction for different TpPa-COF@silica hybrids. Reproduced with permission from ref 102. Copyright 2021 Elsevier. (f) Proposed photoredox mechanism for the heteroaryl–aryl coupling reaction; (g) repeating experiment of P-BT-DEA-CO_2_ for 24 h five cycles. Reproduced with permission from ref 57. Copyright 2018 Wiley-VCH.

#### 3.2.2 Photoreduction Reaction

The photoreduction reaction is a direct utilization of the electrons generated by photoinduced charge separation in organic chemical reactions. Yang reported the photoreductive dehalogenation of α-bromoacetophenone to acetophenone by using TpPa-COF@silica hybrid as a catalyst under a blue light irradiation (30 W, 400–450 nm) in ethanol (Figure 10d).^102^ In this system, Hantzsch ester (HE) not only applied as a hole sacrificial agent but also donated a proton to the α-carbonyl radical formed from α-bromoacetophenone and an electron to produce acetophenone. The hydroxyl groups on the surface of TpPa-COF@silica hybrid NPs of C/S-0.65 might form the hydrogen bond with HE to accelerate the proton donation rate, resulting in a better photoreduction efficiency than that of TpPa-COF NPs (Figure 10e). Similarly, 4-nitrophenol (4-NP) could be photoproduced to 4-aminophenol (4-AP) under a 1 h white light illumination using P-BT-DEA-CO_2_ as the catalyst and sodium borohydride as the hydrogen and electron donor.^57^

#### 3.2.3 Photoredox Reaction

The photoredox catalysis reaction involves both photooxidation and photoreduction processes. Zhang and co-workers reported that P-BT-DEA-CO_2_ could be applied as a photocatalyst for the photoredox synthesis of arylimidazole derivatives by heteroaryl–aryl coupling of caffeine and aryldiazonium tetrafluoroborate (ADTFB) with a yield of 85.2% after exposure of white LED light (power: 0.07 W cm^–2^, λ > 420 nm) for 24 h.^57^ The proposed reaction mechanism suggested that the aryl radical was first formed by electron reduction of ADTFB, which could couple with caffeine to generate a radical. This radical was then oxidized by a hole and deprotonated to achieve the final product. Thus, the photogenerated electron and hole participated in this photoinduced reaction process (Figure 10f). In the repeated reaction, P-BT-DEA-CO_2_ represented good reaction activity without a conversion efficiency loss after five cycling tests (Figure 10g). Another kind of arylimidazole derivative has also been prepared by the photoredox reaction of the CTF-2BT catalyst using *o*-phenylenediamine and benzaldehyde in a yield of 98% after 8 h irradiation.^103^

### 3.3 Hydrogen Evolution

The combustion of fossil fuels produces greenhouse gases, such as carbon dioxide, which exacerbate the greenhouse effect. Hydrogen represents an efficient clean and energy source for industrial energy conversion. Photocatalytic water splitting for hydrogen production directly utilizes solar energy to decompose water into hydrogen with the assistance of a photocatalyst.^109^ This technology originated in 1972, but its main challenge lies in the narrow light absorption band in inorganic catalysts, resulting in a low hydrogen production efficiency. Recently, organic semiconductors with the tunable optical properties have been approved as the promising candidates for the photocatalytic hydrogen evolution.^110^ Traditional organic semiconductors are hydrophobic, and the high water–polymer interfacial energy would limit the production of hydrogen via suppressing electron transfer. Thus, introduction of hydrophilic groups into organic semiconductors via covalent or noncovalent bonds would improve their interfacial behavior and enhance the exciton transfer and dissociation to the surface of catalysts.^27^ Summary of representative photocatalytic H_2_ generation performance using CPNs is listed in Table 2.

**Table 2:** Performance Summary of Representative Photocatalytic H_2_ Generation Using CPNs

sample	morphology (size)	cocatalyst	sacrificial agent	light source	HER (mmol h^–1^ g^–1^)	AQY (%)	refs
PTB7-Th/EH-IDTBR	NPs (150 nm)	Pt (10 wt %)	ascorbic acid	350–800 nm (Xe)	64	6.2 (700 nm)	^27^
PFNDPP-Br	-	Pt (3 wt %)	ascorbic acid	>420 nm (Xe)	11.16	0.44 (650 nm)	^47^
PFBr-PhCN	small fibrous NPs (400 nm)	Pt (3 wt %)	ascorbic acid	>420 nm (Xe)	15.32	0.42 (450 nm)	^50^
PBDTBT-7EO	NPs (5.9 nm)	Pt (3 wt %)	ascorbic acid	>300 nm (Xe)	15.9	0.3 (600 nm)	^59^
PDPP3B–O4	layer-like structure	Pt (1 wt %)	triethanol-amine	>400 nm (Xe)	5.53	5.76 (450 nm)	^60^
PS-PEG5	NPs (83 nm)	Pt (3 wt %)	ascorbic acid	>420 nm (Xe)	11.6	2.92 (405 nm)	^61^
P(BTOEGL-2F2T)	NPs (175 nm)	-	ascorbic acid	>420 nm (Xe)	2.6	2.2 (600 nm)	^62^
P1/P2/H2ase Pdots	NPs (50–120 nm)	methyl viologen (5 mM)	triethanol-amine	420–750 nm (LED)	88.46	1.1 (405 nm)	^63^
P-HEG-10	NPs (1.1 μm)	Pt (5 wt %)	triethyl-amine	>420 nm (Xe)	10.8	18.19 (420 nm)	^69^
PFBT Pdots	NPs (30–50 nm)	-	ascorbic acid	>420 nm (LED)	8.3	0.5 (445 nm)	^75^
PFODTBT Pdots	NPs (30–40 nm)	-	ascorbic acid	>420 nm (LED)	50	0.6 (550 nm)	^76^
PFTBTA-PtPy Pdots	NPs (80 nm)	-	triethyl-amine	>420 nm (LED)	7.34	0.27 (420 nm)	^78^
PFTFQ-PtPy15 Pdots	NPs (20 nm)	-	diethyl-amine	>420 nm (LED)	12.7	0.4 (515 nm)	^79^
D1/D2/ITIC ternary Pdots	NPs (120 nm)	Pt (6 wt %)	ascorbic acid	>420 nm (LED)	60.8	7.1 (700 nm)	^81^
PFODTBT Pdots	hollow NPs (50 nm)	-	ascorbic acid	>420 nm (LED)	18.1	-	^83^
	hollow NPs (70 nm)				4.6		
	hollow NPs (90 nm)				2.6		
HF-CP_10_-Pdots	NPs (69 nm)	-	ascorbic acid	>420 nm (Xe)	0.84	0.9 (500 nm)	^84^
PTTPA/PFTBTA Pdots	NPs (29 nm)	Pt (3 wt %)	ascorbic acid	350–780 nm (Xe)	43.9	-	^88^
P10-e	NPs (156 nm)	-	triethyl-amine	>420 nm (Xe)	14.52	5.8 (420 nm)	^97^
e-TpPa-COFs	spheres (220 nm)	Pt (0.5 wt %)	ascorbic acid	>300 nm (Xe)	45.8	12.6 (420 nm)	^100^
	bowls (1.2 μm)				15.4	-	
	fibers (D: 100–150 nm; *L*: >4 μm)				5.5	-	

#### 3.3.1 CNPs with Homogeneous Core

Tian et al. prepared PFBT NPs for the photocatalytic hydrogen evolution reaction (HER) with an initial rate constant of 45 mmol h^–1^ g^–1^ under a LED light (λ > 420 nm, 17 W), which was 5 orders of magnitude from the PFBT powder.^75^ These results suggested that micellization of the CP can enlarge the surface area and facilitate the charge separation to increase their catalytic activity. However, the performance of PFBT NPs would be deactivated after 1 h of photocatalytic reaction, which may be attributed to the aggregation of these NPs. The energy levels of CPs could be facilely adjusted to improve the photocatalytic hydrogen generation.^62,78,79,97^ Most of the CPs were synthesized by Pd-catalytic cross-coupling polymerization. Thus, the influence of residual Pd in the polymer substrates as a cocatalyst on the photocatalytic performance cannot be ignored. In addition, metallic platinum is often used as the cocatalyst to improve the photocatalytic hydrogen ability.^47,50,59,61,69^ The highest performance for photocatalytic hydrogen evolution was achieved by using PFODTBT-based Pdots 2 (Figure 11a).^76^ The initial HER rate constant of 50 ± 0.5 mmol h^–1^ g^–1^ was obtained in water with the hole sacrificial agent of ascorbic acid (AA) under a LED lamp (λ > 420 nm, 17 W). Owing to the electron acceptor of BT, PFODTBT-based Pdots 2 exhibited a higher apparent quantum yield (AQY) of 0.6% at 550 nm (Figure 11b). In the isotopic labeling experiment, the D_2_ was the main product during the photocatalytic process in D_2_O and NaOD reaction condition, suggesting that the H_2_ came from proton in water rather than that in AA (Figure 11c).

**Figure 11:**
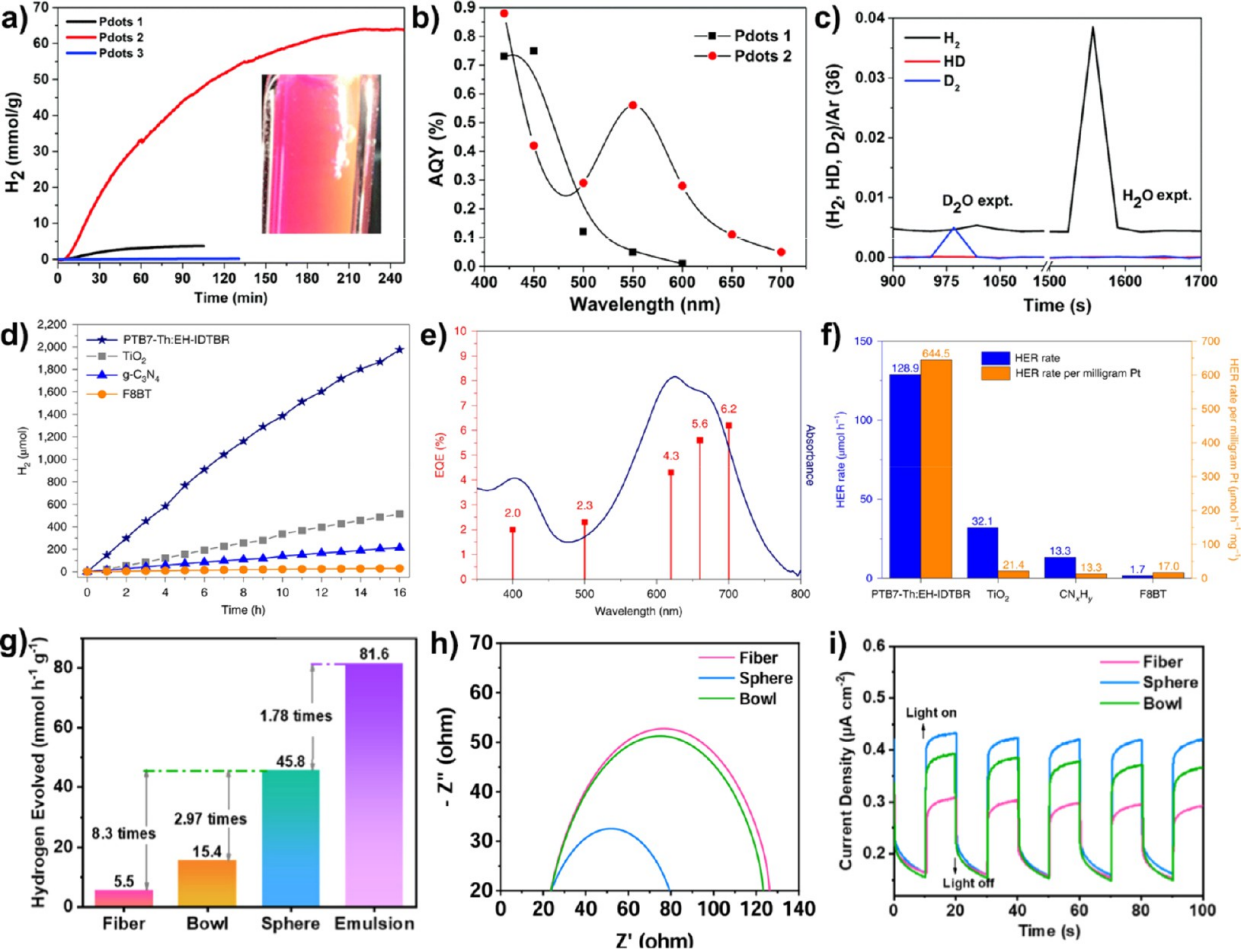
(a) Photoinduced hydrogen generation of Pdots 1 (PFBT), Pdots 2 (PFODTBT), and Pdots 3 (P8T2). (b) AQY as a function of wavelength for Pdots 1 and Pdots 2. (c) Mass spectra of isotopic labeling products. Reproduced with permission from ref 76. Copyright 2017 Royal Society of Chemistry. (d) H_2_ evolution versus time; (e) EQE of PTB7-Th/EH-IDTBR as a function of wavelength. (f) H_2_ evolution rates of the PTB7-Th/EH-IDTBR heterojunction and other controlled groups. Reproduced with permission from ref 27. Copyright 2020 Springer Nature. (g) HER rate of e-TpPa-COF with different shapes. (h) Nyquist plots and (i) transient photocurrent responses for fiber, sphere, and bowl of e-TpPa-COF. Reproduced with permission from ref 100. Copyright 2023 American Chemical Society.

#### 3.3.2 CNPs with Heterojunction Core

Apart from the construction of a D–A-type CP, the optical and electronic properties of these materials can also be tuned via the formation of D/A heterojunction NPs. McCulloch et al. reported that PTB7-Th/EH-IDTBR heterojunction NPs with the mass ratio of 3:7 exhibited the HER rate of 64.43 ± 7.02 mmol h^–1^ g^–1^ in water using AA as hole sacrificial agent and 10% Pt as a cocatalyst under a 300 W Xe lamp with illumination wavelength between 350 and 800 nm (Figure 11d).^27^ The EQE of these NPs were found to be 6.2% at the wavelength of 700 nm (Figure 11e). This unique hydrogen generation performance was superior to other reported photocatalysts (Figure 11f), which was attributed to the strong π–π interaction between PTB7-Th and EH-IDTBR for improving the charge generation inside the NPs. The panchromatic ternary heterojunction NPs was constructed to achieve broad visible-light absorption with high EQE values.^81^ The photocatalytic HER rate of ∼35 mmol h^–1^ g^–1^ was obtained under visible-light (λ > 420 nm, 50 mW cm^–2^) irradiation by using AA as a hole sacrificial agent and 1% Pt as a cocatalyst. The heterojunction NPs were also prepared by using amphiphilic copolymers containing the CP segment as a surfactant.^88^ The electron transfer from NPs to the Pt cocatalyst was enhanced, resulting in a good photocatalytic HER rate of 43.9 mmol h^–1^ g^–1^ under the visible-light illumination (780 > λ > 380 nm, AM 1.5G). By replacing with [FeFe]-hydrogenase (H2ase) as the cocatalyst, the photocatalytic hydrogen generation with a rate constant of 88.46 mmol h^–1^ g_H2ase_^–1^ was obtained under the white light (50 mW cm^–2^, 420–750 nm) in the presence of 10% triethylamine.^63^

#### 3.3.3 Effect on Size of CPNs

The size of CPNs has a great influence on the activity of photocatalytic hydrogen generation. Tian and co-workers prepared the nature-mimicking hollow NPs using PFODTBT as the photoactive material.^83^ They found that the NPs with the size of 50 nm reached the HER rate of 18.1 mmol h^–1^ g^–1^ under the visible-light irradiation (50 mW cm^–2^, > 420 nm), which was 3.93 and 6.96 times higher than that of NPs with the size of 70 and 90 nm, respectively. These results demonstrated that the reduced size of NPs could increase the catalytic site exposure and improve the light capture ability in the hollow structure. The hydrophilic properties of the side chain have a great effect on the NP size of CPNs. Huang et al. prepared poly­(benzodithiophene-*alt*-difluorobenzthiadiazole) (PBDTBT) containing the PEO side chain with different lengths. PBDTBT-7EO with the longest PEO side chain in water formed the NPs with the smallest size of 5.9 nm, which exhibited the highest photocatalytic performance with a HER of 39.75 mmol h^–1^, which was 2.20 and 88.33 times higher than that of PBDTBT-4EO (18.03 mmol h^–1^) and PBDTBT-C6C10 (0.45 mmol h^–1^).^59^ The hydrophilic side chains of CPs play a key role in the formation of NPs with small size to decrease the interfacial energy between the catalyst and reactant, resulting in the enhanced photocatalytic HER activity.

#### 3.3.4 Effect on the Morphology of CPNs

Jin et al. constructed TpPa-COF NPs with controlled morphologies like fiber, bowl, and sphere.^100^ They found that the sphere NPs exhibited a photocatalytic HER rate of 45.8 mmol h^–1^ g^–1^ under the Xe lamp with a cutoff filter (AM 1.5 G, 100 mW cm^–2^), which was 2.97 and 8.3 times higher than that of bowl and fiber shape, respectively, confirming their instantaneous photocurrents (Figure 11g). The electrochemical impedance spectra further indicate that the charge migration and separation in spheres are faster than those of fibers and bowls, originating from the smaller size of NPs for the short charge transfer distance and the improved light absorption (Figure 11h). Moreover, Figure 11i confirms that the photocurrent (0.42 mA cm^–2^) of the spheres was higher than those of the bowls (0.38 mA cm^–2^) and fibers (0.30 mA cm^–2^). Owing to the cationic surface of NPs, TpPa-COF films were obtained on the conductive indium tin oxide substrate via an electrophoretic deposition technique, which showed a HER rate of 7.53 mmol m^–2^ using Pt as the cocatalyst (Figure 11j).

### 3.4 CO_2_ Fixation

#### 3.4.1 CO_2_ Reduction to Value-Added Chemicals

Photocatalytic CO_2_ reduction converting CO_2_ into value-added chemicals by using solar energy has been approved as a promising sustainable and green method to alleviate the energy crisis and reduce the CO_2_ concentration in the atmosphere.^111^ Value-added products with higher energy density and higher market value have attracted extensive research interest. Chen and co-workers prepared the P3HT/GO hybrid for photocatalytic CO_2_ reduction to methanol and acetaldehyde.^72^ The selectivity of products could be readily controlled by the content of P3HT in the P3HT/GO hybrid system. The photocatalytic performance of the hybrid system was higher than that of pure GO due to the improvement of the charge transfer and broad light absorption band.

In search of an efficient strategy to utilize the sunlight to drive chemical conversion, the development of biohybrid photoactivation has gained increasing attention as a novel, sustainable strategy for synthetic chemistry, which offers many advantages including high efficiency, stereoselectivity, and mild reaction condition.^112^ Wang and co-workers developed a p–n heterojunction (PFP/PDI) containing a cationic perylene diimide derivative (PDI) and a cationic poly­(fluorene-*co*-phenylene) derivative (PFP), which served as the photosensitizer for charge generation and separation under the simulated sunlight (Figure 12a).^113^ The electron could efficiently transfer to *Moorella thermoacetica* to synthesize acetic acid from CO_2_, when it was coated on the surface of bacteria by electrostatic and hydrophobic interactions. After triggering the Wood–Ljungdahl pathway, the accumulated acetic acid yield of 0.63 mM was achieved after 3 days of illumination with a power density of 5.0 mW cm^–2^ using cysteine as the electron donor (Figure 12b). Furthermore, *M. thermoacetica* was propagated to 300% during the 3 days of photosynthesis, suggesting that the PFP/PDI showed good biocompatibility to bacteria (Figure 12c).

**Figure 12:**
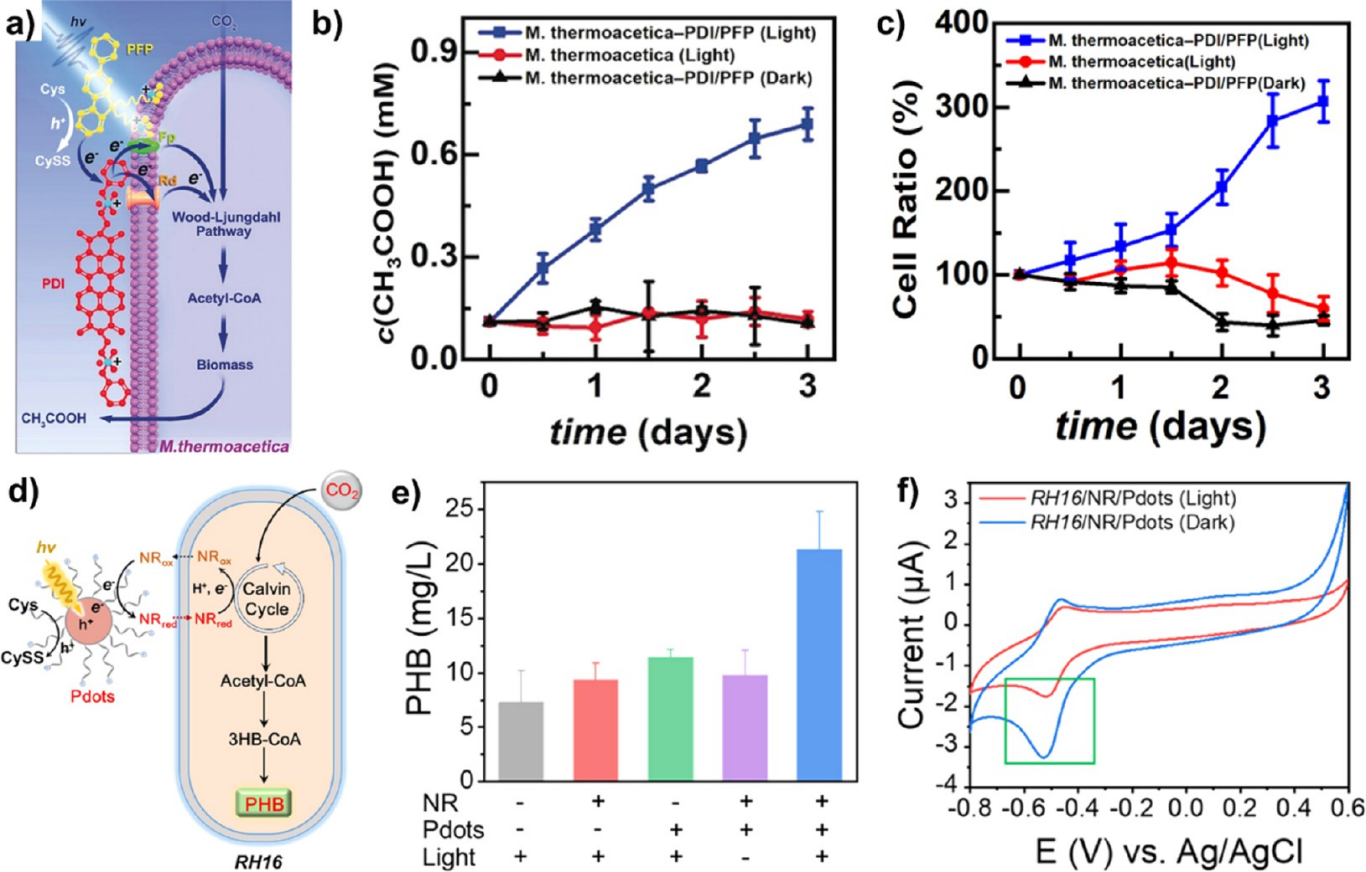
(a) Proposal photocatalytic process of PDI/PFP for CO_2_ conversion to acetic acid using *M. thermoacetica*. (b) The production of acetic acid using PDI/PFP/*M. thermoacetica* (0.63 mM, 0.2 OD_600_ of *M. thermoacetica*) for 3 days with an alternating light–dark cycle. (c) Cell viability of *M. thermoacetica* in varying photocatalytic conditions. Reproduced with permission from ref 113. Copyright 2020 Wiley-VCH. (d) Schematic illustration of RH16/neutral red (NR)/Pdots for photocatalytic CO_2_ to PHB. (e) PHB photocatalytic generation in different conditions (0.5 OD_600_ of RH16, 0.02% cysteine, NR: 20 μmol L^–1^ and 4 μg mL^–1^ Pdots). (f) CVs of RH16/NR/Pdots with and without light irradiation. Reproduced with permission from ref 85. Copyright 2023 American Chemical Society.

#### 3.4.2 CO_2_ Reduction to a Polymer

Recently, Wang et al. reported the preparation of PFODTBT-based NPs as the photosensitizer for the construction of a ternary synergistic biohybrid photoactivation system containing neutral red (NR) as the electron shuttle and *Ralstonia eutropha* H16 (RH16) as the bioreactor (Figure 12d).^85^ Under a xenon illumination with an AM 1.5G filter and 2.5 mW cm^–2^, the reduction of nicotinamide adenine dinucleotide phosphate from the photogeneration of electron could activate the Calvin cycle in the RH16 for the photosynthesis of poly­(3-hydroxybutyrate) (PHB) from CO_2_. The photosynthesis could cumulate the PHB concentration of the ternary biohybrid of up to 21.3 ± 3.78 mg L^–1^, which was nearly three times higher than that of RH16 (Figure 12e). The cyclic voltammetry curves demonstrated that the photoreduction of NR participated in the biosynthesis of PHB (Figure 12f).

### 3.5 Medical Therapy

Phototherapeutics is a promising noninvasive treatment for cancer because of its spatial and temporal control, low drug resistance, and possibility of precision dosing.^114^ CP-based NPs possess good water dispersion, efficient charge generation and separation, and structural stability, which have been demonstrated as the unique platform for cancer phototherapy. Photodynamic therapy (PDT) is a type of mature clinical treatment through the production of reactive oxygen species (ROS) to kill cancer cells and fungus.^115^ The CPNs as the photosensitizers absorb the light energy and are promoted into an excited triplet state, which undergoes the photochemical reaction with intracellular oxygen to prepare cytotoxic singlet oxygen (^1^O_2_). Wang and co-workers reported the preparation of thiol-functionalized cationic poly­(fluorene-*co*-thiophene) (PFT-SH), which could be cross-linked into large aggregation in the cancer cells by in situ forming disulfide bonds to improve the drug efficacy.^116^ Both HeLa and A549 cells were effectively inhibited via PDT after the 680 nm-light irradiation (4 mW cm^–2^) for 30 min. To improve the deep penetration of traditional PDT, Qiu and co-workers constructed a novel type of amphiphilic unimicelles containing a hyperbranched CP core.^67^ After grafting of Ce6 on the surface of unimicelles, the toxic ^1^O_2_ was generated efficiently via a synergistic strategy of two-photon FRET and photothermal effect under 800 nm-light irradiation with 0.7 mJ per pulse (Figure 13a), resulting in high inhibition proliferation of HeLa cells with the lowest IC_50_ value of 3.95 ± 0.21 μg mL^–1^ (Figure 13b). The in vivo antitumor activity studies demonstrated that these unimicelles not only exhibited good selective accumulation in the tumors by the EPR effect but also achieved the highest tumor inhibitory rate (TIR) of 87.1 ± 1.7% against the HeLa tumor among all therapeutic groups after the effective two-photon PDT for 20 days (Figure 13c). The hypoxia microenvironment in cancer cells also restricts the treatment efficiency of PDT, Wang et al. constructed a biomimetic NPs by enveloping hemoglobin (Hb)-linked conjugated polymeric NPs into the liposome.^117^ Using luminol as the chemiluminescence resource, PDT against HeLa cells was carried out without external light. The Hb was used as the oxygen carrier to improve the PDT efficiency. Furthermore, the generation of ROS could trigger the release of chlormethine for the synergy of PDT and chemotherapy, leading to a better tumor cell inhibition effect.

**Figure 13:**
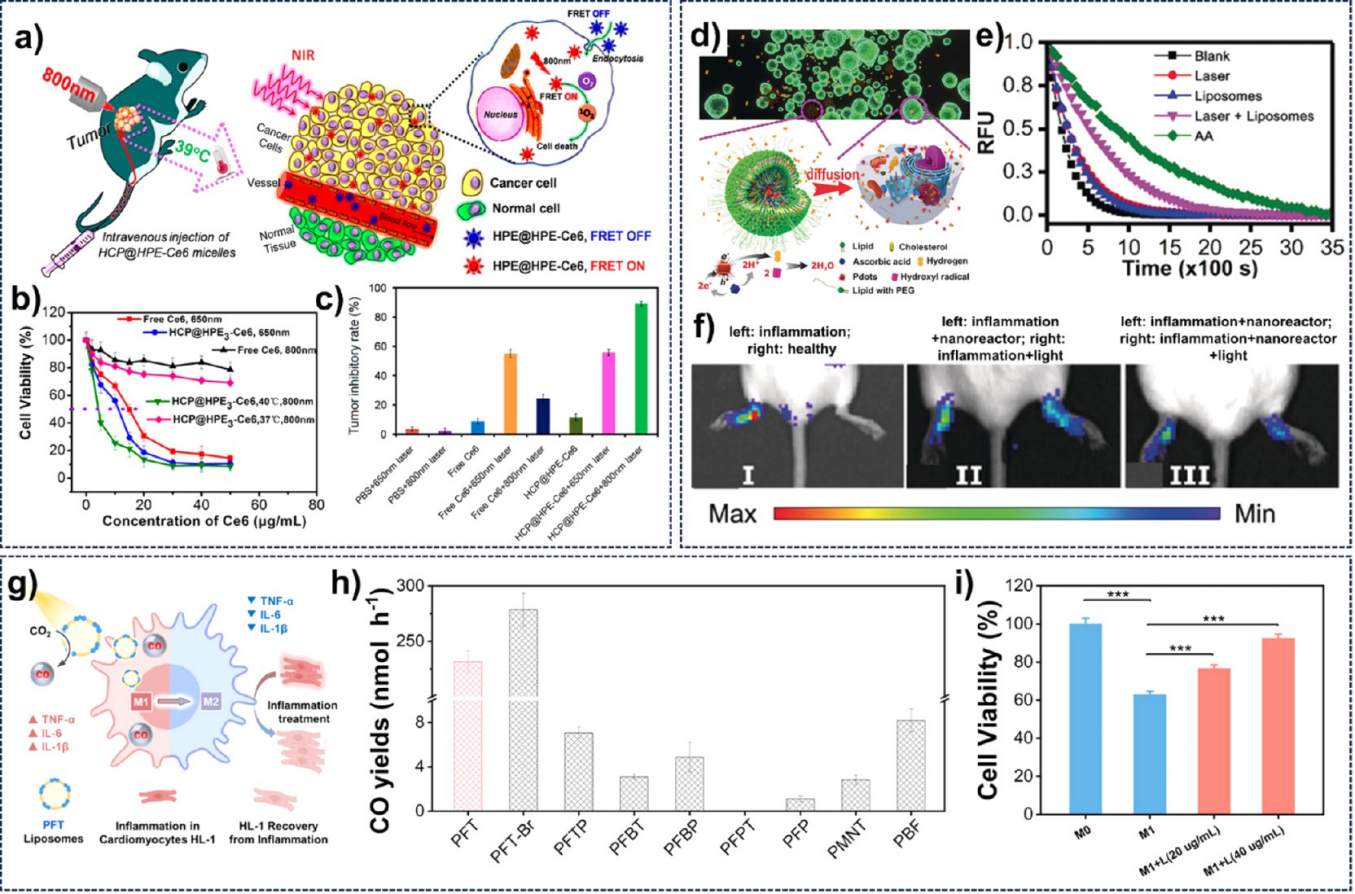
(a) Proposal PDT anticancer activity of HCP@HPE-Ce6 micelles. (b) In vitro photocytotoxicity of different treatment groups. (c) TIR pretreated with different treatment groups. Reproduced with permission from ref 67. Copyright 2016 American Chemical Society. (d) Schematic illustration of nanoreactor for intracellular photocatalytic H_2_ to antioxidation. (e) Antioxidant ability of different treatment conditions evaluated from the PL time of the fluorescent probe. (f) PL images of LPS-induced inflamed paws pretreated with different therapeutic conditions. Reproduced with permission from ref 87. Copyright 2019 Wiley-VCH. (g) Proposal photocatalytic CO_2_-to-CO conversion for immune regulation in inflammation therapy. (h) CO production rate of PFT and other six CP NPs in acetonitrile. (i) HL-1 cell viability after coculture experiments. Reproduced with permission from ref 122. Copyright 2024 American Chemical Society.

As mentioned above, gaseous H_2_ and carbon monoxide (CO) could be efficiently produced by the photocatalytic reduction reaction from water and CO_2_, respectively, which has been applied for the phototherapeutics. Excessive production of ROS is commonly observed in the initial stages of many pathological conditions. Delivering reductive H_2_ to counteract ROS overexpression is highly desirable.^118^ Zhang and co-workers prepared the CNPs for in situ photocatalytic production of H_2_, which penetrated the liposome bilayer of the cell to act against ROS in the inflamed tissues (Figure 13d).^87^ Under the visible light with a > 420 nm cutoff filter by a 300 W xenon lamp, PFODTBT-based CNPs as a photocatalyst could efficiently generate the electron for the reduction reaction of H^+^ into H_2_ without the cocatalyst in the aqueous solution. Thus, the photoinduced generation of H_2_ could remove the radical ^•^OH from the Fenton reaction, resulting in a long PL time of the fluorescent probe. A similar phenomenon was also observed using AA as the reducing agent (Figure 13e). As shown in Figure 13f, the luminescent images suggested that the oxidative stress in the inflamed paw was mitigated obviously by pretreatment with the conjugated NPs under laser irradiation, leading to a low ROS-responsive fluorescence intensity.

Cancer exhibits the typical hypoxia condition with high CO_2_ concentration level, which could be in situ photocatalytically converted to CO in the presence of the photosensitizer and light source via the photoreduction reaction of CO_2_-to-CO. Some research works demonstrate that CO could promote the polarization of macrophages to inhibit the pro-inflammatory cytokines secretion, resulting in the mitigation of the progression of disease.^119–121^ Wang and co-workers investigated nine cationic CPs for photocatalytic CO_2_-to-CO conversion for the inhibition of the pro-inflammatory cytokines secretion (Figure 13g).^122^ They found that PFT exhibited photocatalytic CO generation with reaction ratios of 231 nmol h^–1^ and 46 nmol h^–1^ in acetonitrile and aqueous solutions, respectively, under white light (xenon lamp, AM1.5, 35 mW cm^–2^) (Figure 13h). The CO selectivity of PFT in water reached 88%. After loading into the liposomes, these NPs could generate in situ CO from intracellular CO_2_ under white-light irradiation, significantly suppressing the apoptosis of the activated macrophages via anti-inflammatory treatment (Figure 13i). Apart from the photocatalytic generation of a signaling molecule, the direct photogradation of biomolecules could also be the effective approach for disease treatment. The same research group reported the photocatalytic degeneration of nicotinamide adenine dinucleotide (NAD^+^) by using cationic PFP as the photosensitizer.^123^ Under white light illumination with 50 mW cm^–2^, the continuous depletion of NAD^+^ disturbs coenzyme recycling, leading to mitochondrial dysfunction and ultimately promoting apoptosis in 4T1 tumor cells.

## 4 Challenges and Perspectives

In this review, we have systematically reviewed the significant achievements of the CPNs for photocatalytic application aimed at addressing the major limitations of the hydrophobic character of traditional CPs. The micellization of CP with the hydrophilic functional groups has been exploited as a promising avenue to improve the photocatalytic performance via various construction strategies, including the covalent chemical reaction with hydrophilic small functional groups or polymers, supramolecular interaction with small surfactants or diblock copolymers, and in situ polymerization with soft or hard templates. Based on reported studies, these CPNs have several advantages as follows: (1) high dispersion stability in aqueous solution via improving their surface wettability; (2) enhancement of the light absorption by minimizing the size of CPNs; and (3) promoting their electron transfer to the surface of the photocatalyst by restricting the charge/electron recombination. Thus, these unique photocatalytic properties endow them with wide applications in the fields of organic pollutant degradation, chemical transformations, hydrogen evolution, CO_2_ fixation, and medical therapy.

In spite of significant progress in CPNs for the photocatalytic applications achieved, this field is still in its infancy, and huge challenges still should be addressed toward both academic research and commercial applications. To solve these critical issues, some improvements below need to be considered: (1) the visible-light absorption exhibits finite light penetrability, and most of CPNs have strong light scattering effect, which would limit their photocatalytic performance in a wide range of applications. NIR light covers 50% of the solar spectrum and exhibits excellent penetrating properties, which provides a novel approach to enhance photocatalytic performance.^124–126^ Thus, it is a crucial requirement for developing CPN photocatalysts with NIR light absorption via rational structural design of D–A-type CPs. (2) The ordered stacking of the CP in the CNPs could be beneficial to the photoinduced exciton transfer in the micelles, which enhance their photocatalytic performances.^127,128^ Construction of a novel kind of CNPs as photocatalysts via living CDSA is highly desirable.^35,64^ (3) Previous works reported that the size and morphology of CPNs have the influence on the photocatalytic HER.^83,100^ Establishing the systematic relationship between the size and morphology of CPNs and their photophysical properties is to help better understand the mechanism of photocatalytic performance. (4) Exploring CPs for photocatalytic CO_2_ and nitro chemicals conversion into high-value products has received great attention by scientists.^129,130^ The development of new CPNs with rich photoredox activities for efficient photochemical energy conversion will boost its competitiveness in applications. (5) Although high-performance photocatalysts are achieved in batch reactors, the yields of targeted products are still limited. Currently, continuous-flow reactors for photosynthesis have been reported, in which the photocatalytic efficiencies are greatly enhanced.^131,132^ CPNs with good solubility facilitate the solution process for photocatalytic layers for industry-scale photosynthesis production using flow reactors. It is believed that CPNs will emerge as an increasingly attractive class of photocatalysts for both academic and practical applications in the future.
